# Are Dietary Proteins the Key to Successful Body Weight Management? A Systematic Review and Meta-Analysis of Studies Assessing Body Weight Outcomes after Interventions with Increased Dietary Protein

**DOI:** 10.3390/nu13093193

**Published:** 2021-09-14

**Authors:** Thea Toft Hansen, Arne Astrup, Anders Sjödin

**Affiliations:** Department of Nutrition, Exercise and Sports, Section for Obesity Research, Faculty of Science, University of Copenhagen, 1958 Frederiksberg C, Denmark; ast@nexs.ku.dk (A.A.); amsj@nexs.ku.dk (A.S.)

**Keywords:** appetite, obesity, overweight, satiety, weight loss

## Abstract

The primary aim was to systematically review the current evidence investigating if dietary interventions rich in protein lead to improved body weight management in adults with excessive body weight. The secondary aim was to investigate potential modifying effects of phenotyping. A systematic literature search in PubMed, Web of Science, and Cochrane Library identified 375 randomized controlled trials with 43 unique trials meeting the inclusion criteria. The Cochrane collaboration tool was used for a thorough risk of bias assessment. Based on 37 studies evaluating effects of dietary protein on body weight, the participants with increased protein intake (ranging from 18–59 energy percentage [E%]) were found to reduce body weight by 1.6 (1.2; 2.0) kg (mean [95% confidence interval]) compared to controls (isocaloric interventions with energy reduction introduced in certain studies). Individuals with prediabetes were found to benefit more from a diet high in protein compared to individuals with normoglycemia, as did individuals without the obesity risk allele (AA genotype) compared to individuals with the obesity risk alleles (AG and GG genotypes). Thus, diets rich in protein would seem to have a moderate beneficial effect on body weight management.

## 1. Introduction

Many different diets are proposed for prevention and treatment of overweight and obesity. In several popular diets, the overall strategy is to manipulate macronutrient composition within the whole diet and to focus on limiting or increasing one macronutrient [1,2,3]. Classic theories posit that macronutrient composition of a diet or a meal affects appetite via homeostatic mechanisms, triggering or inhibiting energy intake to ensure that the intake of energy or nutrients matches bodily needs [4]. Accordingly, the amino static theory suggests that there is a nutrient-specific hierarchy of satiating power, with protein having a more satiating power than carbohydrate, which is in turn more satiating than fat [5]. Several mechanisms have been suggested to contribute to the satiating value of dietary protein, including that the presence of amino acids or peptides in the gastrointestinal (GI) tract contributes as ligands for receptors in the small intestine, triggering release of anorexigenic signaling molecules, and is thereby involved in the gut-brain axis [6,7,8,9,10,11]. As appetite is one of the numerous influences that determine energy intake, it has been argued that increased intake of dietary protein is beneficial for body weight management. Furthermore, there is a fairly consistent body of evidence suggesting that diets higher in protein increase energy expenditure due to an increased thermic effect [12]. This may, at least in part, be explained by the fact that the body has a limited capacity for storing amino acids or protein, and thus needs to metabolize dietary protein immediately, which includes processes requiring energy [13,14].

The positive effects of dietary protein on body weight management have been observed in large long-term randomized controlled trials [15]. However, a previous meta-analysis assessed low-carbohydrate/high-protein diets effects on body composition during energy restriction, and the amount of dietary protein was not found to be linked to the degree of changes in body weight. However, there was a tendency for reported protein intake to predict changes in body fat [16]. Several studies investigating the effects of dietary protein on appetite have failed to control for energy density, which renders results showing a more satiating power of protein per se. Therefore, it remains unclear whether dietary protein is beneficial for body weight management in the context of overweight. Furthermore, recent findings suggest that successful body weight management with specific diets varying in macronutrient content may be highly dependent on specific phenotypes including degree of glucose tolerance, appetite and eating behavior characteristics as well as different genotypes [17,18,19,20].

On this basis, the primary aim of this systematic review and meta-analysis was to summarize the current literature, to investigate if dietary interventions rich in protein lead to improved body weight management in adults with overweight or obesity. The secondary aim was to investigate potential modifying effects of physiological or behavioral phenotypes.

## 2. Materials and Methods

A comprehensive review protocol was prepared in collaboration between the authors in advance of the systematic literature search. The review protocol followed the preferred reporting items for systematic reviews and meta-analyses (PRISMA) guidelines [21] and was used to identify objectives of the review including population, intervention, comparison, outcome, and setting (PICOS), as well as methods for literature search and data extraction. No major deviations to the original protocol occurred and minor updates were recorded with protocol version number and date. The review protocol was not pre-registered.

### 2.1. Data Sources and Search Strategy

The systematic literature searches were conducted in PubMed, Web of Science, and the Cochrane Library. Studies potentially eligible for inclusion and available as of 1 September 2021 were identified. The search strategy was built in PubMed based on screening of medical subject heading (MeSH) term index list, as well as testing numerous combinations of search terms, to obtain the most hits. Similar searches were subsequently conducted in Web of Science and Cochrane Library. The final primary search syntaxes employed for each of the databases are described in Table 1. To identify more recently completed trials, unpublished research, and research reported in grey literature, unsystematic searches were conducted in Google Scholar and ClinicalTrials.gov, and experts within the field were consulted. Finally, reference lists of the included papers were screened. Secondary unsystematic literature searches were also conducted in PubMed and Google Scholar in order to identify more studies, additional to what was identified from the primary searches, investigating potential different effects of specific proteins, peptides, and/or amino acids, as well as physiological or behavioral characteristics (phenotypes) that may cause differentiated effects.

All authors contributed to the search strategies, and ultimately the first author conducted the searches. The first and last authors independently assessed eligibility of the studies based on screening of titles, abstracts, and full texts. All authors contributed to discussions of eligibility.

### 2.2. Inclusion and Exclusion Criteria

Only studies with adult (≥18 years) human populations with overweight or obesity with or without comorbidities were included in the review. Papers were excluded if the study population consisted of athletes, underweight participants, or participants with anorectic diseases. The interventions and comparisons eligible for inclusion included dietary protein compared to other macronutrients (substituting carbohydrate or fat with protein), or only adding more protein to the diet without manipulating carbohydrate or fat. No specific criteria for “high dietary protein” were set, but the studies were required to justify a relative difference. Interventions investigating potential effects of different proteins, peptides, and/or amino acids compared to each other were also included and assessed in a separate meta-analysis. Papers reporting effects of protein recommendations based on phenotyping were also included in the review, but these were not included in the meta-analysis. Certain peptides have been identified as effective in reducing appetite and potentially beneficial for body weight management and are increasingly being used as pharmaceuticals for obesity treatment [22,23,24,25,26]. However, interventions that included pharmaceuticals were excluded, as the focus of this review was on dietary protein. Interventions including changes in exercise was excluded since this may mask potential dietary effects on body weight management. Effects of interventions on body weight changes (weight loss, weight loss maintenance or weight maintenance) were assessed. Thus, interventions could include energy restriction or not, but similar recommended energy restriction was required for the intervention and the controls groups within each study. No acute studies could be included in this review since the outcome was changes in weight, but there were no specific restrictions on intervention duration. All outcomes were required to be assessed under laboratory settings, and only studies including randomized controlled trials were included.

### 2.3. Outcomes and Data Extraction

The primary outcome was body weight change (kg), which was included in the meta-analyses. If available, data from intention-to-treat (ITT) analyses were included in the meta-analyses. Details of the study protocols and methodologies as well as mean ± standard deviation (SD) demographic characteristics (n [male/female], age, and body mass index (BMI)) of the study populations within each of the studies were extracted. These details are summarized, describing the key characteristics, in Table 2. Detailed descriptions of each of the studies are presented in Table S1 of the supplementary material. The intervention and relative daily prescription of macronutrient distribution was calculated based on the quantity of protein per kg of mean baseline body weight if energy percentage (E%) was not reported. High/rich protein diet was defined relative to the control diet in each study. Thus, for certain studies, high/rich protein diet constitutes diets of normal protein content (15–20 E%), but the control diet constitutes diets of low protein content (<15 E%). Diets are referred to as “high protein” vs. “standard protein” throughout the paper. Thus, overlap in E% protein between high protein (HP) and standard protein (SP) exists between papers. Diet characteristics are summarized in Table 2 and detailed descriptions for each of the studies are presented in Table 1.

The first author extracted the data, which was randomly checked by the last author. The investigators of the original studies were contacted if information was lacking. For studies with multiple treatment arms, where certain arms did not meet the inclusion criteria for this review, only data from the arms meeting our inclusion criteria were included.

### 2.4. Risk of Bias Assessment

Criteria for risk of bias were set based on the Cochrane collaboration tool for assessing risk of bias [27]: 1. Random sequence generation (selection bias). 2. Allocation concealment (selection bias). 3. Blinding of participants and personnel (performance bias). 4. Blinding of outcome assessment (detection bias). 5. Incomplete outcome (body weight) data (attrition bias) (including whether or not data from ITT analyses are available). 6. Selective reporting (reporting bias) (reporting differences in body weight outcome between groups or not). 7. Power calculation. 8. Drop out. 9. Compliance. Risk of bias was rated as “low” or “high” according to predefined specifications (see Supplementary Material Table S2 (legend)), or “unclear” if no information on a potential bias was reported.

### 2.5. Meta-Analyses

Random effects meta-analyses were performed to allow for differences in the study populations, treatments etc. The primary analysis included differences in body weight change (kg) between exposures to various interventions with increased protein intake compared to controls (digestible carbohydrate, fiber, fat, no supplementation (no placebo used)). Additionally, analysis of differences in body weight changes (kg) between exposures to specific proteins was conducted to investigate if certain dietary proteins seem more effective for body weight management than others.

If mean and 95% confidence interval (CI) difference in body weight change were not directly reported in the papers, the effect sizes were calculated based on reported changes within each group. If no SD, standard error of mean (SEM), or 95% CI for the differences in body weight change between groups were reported, the 95% CI was imputed based on the mean difference calculated from the changes within each diet group and the average SEM from the other studies [28]. We report the cases where this imputation resulted in a non-significance/significance between groups contrary to the findings in the original paper.

The presence of inter-study heterogeneity was quantified using the I2 statistic evaluating >50% as substantial [28]. Potential sources of clinical and methodological heterogeneity were investigated by sensitivity and subgroup analyses. Sensitivity analyses were performed removing all studies weighing >3.0% in the random effects analysis, as well as based on the risk of bias assessment removing all studies evaluated to have >3 criteria with high risk of bias, or >5 criteria with high risk of bias or unclear. In addition, a priori post hoc subgroup analyses removing studies based on subgroups of risk of bias were conducted, including removal of studies evaluated with high risk of bias due to high drop-out rate or due to low compliance. Additionally, if several comparisons were reported in a study, each comparison was included in the meta-analysis. Thereby, the same study population of the control groups could be represented several times, which may introduce a risk of bias. A sensitivity analysis excluding the studies represented several times was conducted to exclude that these cases affect the overall result of the primary meta-analysis.

Publication bias was assessed by visual inspection of funnel plot and by Egger test to assess asymmetry, as well as by Begg test to assess small study effects [29,30,31].

The meta-analyses were conducted using StataCorp. 2017. Stata Statistical Software: Release 15. College Station, TX: StataCorp LLC. Texas, USA.

## 3. Results

### 3.1. Studies Included

**Figure 1:**
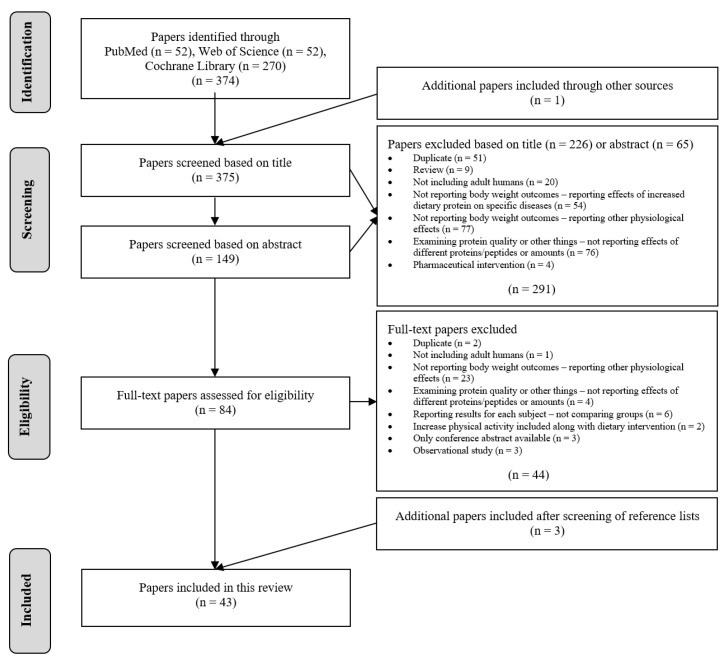
PRISMA flow chart explaining the literature selection process after systematic literature searches in PubMed, Web of Science and Cochrane Library, identifying studies potentially eligible for inclusion and available as of 1 September 2021.

The searches identified a total of 375 publications for screening, of which 291 were excluded based on titles and abstracts. An additional 44 were excluded based on full-text assessments. Three additional papers were included after screening of reference lists of the included papers, resulting in inclusion of 43 papers in total (Figure 1).

### 3.2. Study Characteristics

All studies employed a parallel design of which the majority used a non-blinded approach. The diets were included during a mean of 32 weeks interventions, ranging from 8 to 104 weeks. Interventions primarily included comparisons of dietary protein and digestible carbohydrate totaled 35 unique papers [32,33,34,35,36,37,38,39,40,41,42,43,44,45,46,47,48,49,50,51,52,53,54,55,56,57,58,59,60,61,62,63,64,65,66], of which one also assessed comparison of dietary protein with fat [47], three also assessed different effects from specific proteins (whey protein) [33,36,51], and three investigated effects of personalized protein recommendations based on phenotyping [37,41,59]. Several studies investigated comparisons of dietary protein with fiber (two papers) [67,68], with fat (one paper) [69], or with no supplementation (no placebo used) (two papers) [70,71]. Several studies only compared effects of specific proteins (lupin, animal vs. vegetable protein and glycomacropeptide) and were included only in the secondary meta-analysis (three papers) [72,73,74]. See Table 2 and Supplementary Material Table S1 for information on study features and participant characteristics.

### 3.3. Effects of Dietary Protein on Body Weight Management

**Figure 2:**
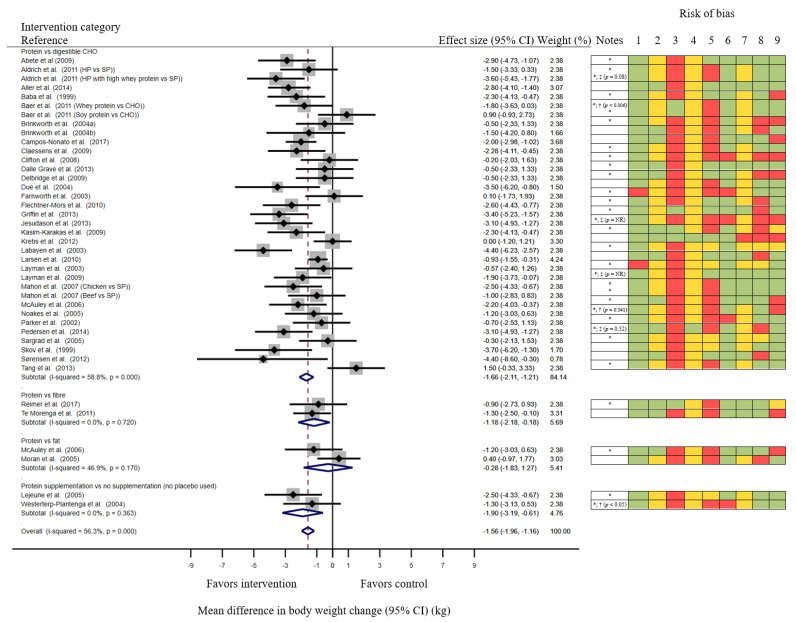
Meta-analysis of mean difference in body weight changes with 95% CI (kg) between exposures to various interventions increasing protein intake compared to controls. CHO, carbohydrate; CI, confidence interval; HP, high protein (30 E%), NR, not reported; SP, standard protein (range: 15–16 E%); *, 95% CI imputed based on the average standard error of mean from the other studies [28]; †, Imputation of 95% CI causes non-significant difference despite significant difference is reported in the original paper; ‡, Imputation of 95% CI causes significant difference despite non-significant difference being reported in the original paper.

Meta-analysis of the 37 studies [32,33,34,35,36,38,39,40,42,43,44,45,46,47,48,49,50,51,52,53,54,55,56,57,58,60,61,62,63,64,65,66,67,68,69,70,71] evaluating effects of dietary protein on body weight changes found that participants exposed to various interventions increasing dietary protein (ranging from 18–59 energy percentage (E%)) reduced their body weight by 1.6 (1.2; 2.0) kg (mean (95% CI)) compared to controls (digestible carbohydrate, fiber, fat or no supplementation (no placebo used); Figure 2). Studies were closely weighed in the random effects meta-analysis, but there was a moderate uncertainty around the estimate (I2 = 56%). A sensitivity analysis was performed removing all studies weighing > 3.0% in the random effects analysis (6 assessments [34,50,62,64,68,69]), resulting in a slight decrease in the heterogeneity and increased estimated effect size (mean (95% CI) body weight reduction compared to controls: 1.7 (1.2; 2.1) kg; I2 = 51%).

The funnel plot showed no sign of asymmetry (Supplementary Material Figure S1). Slight tendencies for asymmetry (mean bias (95% CI): −1.4 (−2.8; 0.1), *p* = 0.06) and small-study effects (Kendall’s tau: 1.8, *p* = 0.07) were indicated by the Eggers and Begg tests, respectively.

The meta-analysis is divided into categories of interventions. The grey marks around the mean from each study indicate the weight of the evidence from each study assessed in a random effects analysis. The blue diamonds summarize the total mean differences for each of the intervention categories, and finally for the overall result with width of the diamonds indicating the 95% CI.

The following criteria for risk of bias were assessed: 1. Random sequence generation (selection bias). 2. Allocation concealment (selection bias). 3. Blinding of participants and personnel (performance bias). 4. Blinding of outcome assessment (detection bias). 5. Incomplete outcome (body weight) data (attrition bias) (including if data from ITT analyses are available or not). 6. Selective reporting (reporting bias) (reporting differences in body weight outcome between groups or not). 7. Power calculation. 8. Drop out. 9. Compliance. Risk of bias was rated as “Low”—indicated as green or “High”—indicated as red according to predefined specifications (see Supplementary Material Table S2 (legend)) or “Unclear”—indicated as yellow if no information on a potential bias was reported.

### 3.4. Evaluation of Risk of Bias

The risk of bias assessments is summarized along with the meta-analysis in Figure 2 and reported in detail in Supplementary Material Table S2. For most of the studies, allocation was described as randomized, but many of the studies lacked detailed description of allocation concealment procedure. Most of the studies were evaluated to give a high risk of biasing the outcome of this review as they were non-blinded, because of obvious differences between intervention and control diets. Additionally, most studies failed to report whether data firstly were assessed blinded by a person not directly involved in the study. Most of the studies reported results from complete case analyses, introducing a risk of bias if the discontinued participants were the ones experiencing no benefit from the interventions. Most of the studies reported comparisons between the groups including significance levels for the differences, but most of the studies failed to report the estimated difference including the variance. For most of the studies 95% CI was therefore imputed based on the mean difference calculated from the changes within each diet group and the average SEM from the other studies. Many of the studies failed to report whether a power calculation was conducted; thus, we could not evaluate whether the preferred sample size was achieved. Nevertheless, many of the studies achieved acceptable completion rates (≤25% drop-out was evaluated as expectable, as most of the studies include long-term interventions) and reported high compliance in both intervention and control groups.

Sensitivity analysis removing all studies evaluated to have >3 criteria with high risk of bias, or >5 criteria with high risk of bias or unclear (i.e., 11 studies [38,43,44,48,52,56,60,63,66,69,71]), resulted in an outcome comparable to the main analysis with mean (95% CI) body weight reduction compared to controls of 1.8 (1.3; 2.2) kg; I2 = 53% in a random effects analysis. The first post hoc subgroup analysis where studies evaluated with high risk of bias due to high drop-out rate were removed (13 studies [40,45,48,49,52,54,57,58,60,61,62,64,69]) showed a mean (95% CI) body weight reduction compared to control of 1.6 (1.2; 2.1) kg; I2 = 52% in a random effects analysis. The second post hoc subgroup analysis where studies evaluated with high risk of bias due to low or unclear compliance were removed (13 studies [35,43,47,48,50,52,54,60,61,62,63,67,68]) showed a mean (95% CI) body weight reduction compared to controls of 1.6 (1.1; 2.1) kg; I2 = 60% in a random effects analysis. Sensitivity analysis removing studies that reported several comparisons (this was only the case for four out of the 37 studies [33,36,46,47]) did not affect the outcome of the meta-analysis (mean (95% CI) body weight reduction compared to controls: 1.6 (1.2; 2.0) kg; I2 = 55%).

### 3.5. Different Effects from Specific Proteins

In the six studies [33,36,51,72,73,74] assessing different effects from specific proteins (whey protein vs. soy or casein, lupin flour vs. wheat flour, animal vs. vegetable protein, glycomacropeptide-enriched whey protein isolae vs. skim milk powder), no specific protein seemed to be superior compared to the matching controls. However, only one [72] of the six studies reported the estimated difference including the variance. Thereby, the variance was imputed for the remaining five studies, introducing a significant risk of bias. Therefore, this evidence was not found sufficient to qualify for a proper meta-analysis, and the results are limited to the Supplementary Material Figure S2 and should be interpreted with caution.

### 3.6. Effects on Body Weight Management of Personalized Protein Recommendations Based on Phenotyping

Two studies were identified that assessed differences between normoglycemic and prediabetic individuals in body weight management [37,59]. Ballesteros-Pomar et al. assessed potential differentiated effects during weight loss. After 16 weeks of energy restriction, they found non-significant mean difference of 1.9 kg in responsiveness to the high protein diet (30 E% protein) vs. the standard protein diet (15 E% protein) between participants of normoglycemia (*n* = 15) and prediabetes (*n* = 21) based on the homeostatic model assessment for insulin resistance (HOMA-IR). Participants with prediabetes consuming the high protein diet (*n* = 10) were found to lose 2.6 kg more than those consuming the standard protein diet (*n* = 11), whereas the participants with normoglycemia lost only 0.7 kg more on the high protein diet (*n* = 8) compared to the standard protein diet (*n* = 7) [37]. Hjorth et al. assessed potential differentiated effects during weight loss maintenance. After a mean weight loss of 10.3 kg in the DIOGENES study, they found a mean (95% CI) difference of 4.4 (1.8; 7.0, *p* = 0.001) kg in responsiveness to the high protein diet (21 E% protein) vs. the standard protein diet (17 E% protein) between participants of normoglycemia (*n* = 225) and prediabetes (*n* = 41) based on fasting plasma glucose. Participants with prediabetes consuming the standard protein diet (*n* = 7) were found to regain 5.8 (3.3; 8.2) kg more than those consuming the high protein diet (*n* = 23), whereas the participants with normoglycemia regained only 1.4 (0.5; 2.4) kg more on the standard protein diet (*n* = 88) compared to the high protein diet (*n* = 91) [59].

One study assessing differences in body weight management based on genotyping was identified [41]. Stocks et al. assessed potential differentiated effects during weight loss maintenance. After a mean weight loss of 11.0 kg in the DIOGENES study, they found that the rs987237 single nucleotide polymorphism (SNP) of the transcription factor AP-2 beta gene (TFAP2B) interacted with protein intake in relation to weight loss maintenance (*p* = 0.047). The authors found that the participants without the obesity risk allele (AA genotype) (*n* = 251) regained 1.5 (0.7; 2.2) kg (mean (95% CI)) on the standard protein diet (13 E% protein), whereas they lost an additional 0.9 (0.0; 1.7) kg on the high protein diet (26 E% protein). This beneficial effect of the high protein diet on body weight was not seen among carriers of the obesity risk alleles (AG (*n* = 110) and GG genotypes (*n* = 12)). Stocks et al. also found that the participants consuming the high protein diet regained 1.8 (0.0; 3.7) kg more body weight per obesity risk allele (G allele) compared to participants consuming the standard protein diet [41].

## 4. Discussion

Dietary protein seems to have a more beneficial effect on body weight management in individuals with overweight or obesity than carbohydrate. This meta-analysis also indicates, although less strongly, that dietary protein is more beneficial for body weight management than dietary fiber, as well as when protein is additionally supplemented to the diet, whereas no effect is seen when compared with fat. However, the number of studies substituting dietary protein with alternatives other than carbohydrate is small. There does not seem to be sufficient evidence to determine whether specific proteins are linked to a greater effect than others, but this may again be due to the limited number of studies fulfilling the criteria for this review. The effect size of dietary protein in body weight management may also be dependent on specific phenotypes, where individuals with prediabetes may benefit more compared to individuals with normoglycemia. Furthermore, individuals without the obesity risk allele (AA genotype) may benefit more compared to individuals with the obesity risk alleles (AG and GG genotypes).

### 4.1. Efficacy of Higher Dietary Protein Intake for Body Weight Management

The analyses in this review are limited to the effects of higher dietary protein on body weight changes because studies investigating potential effects on changes in body composition are scarce. Differences in efficacy may be observed dependent on whether the effects on body weight or fat mass changes are evaluated, which is indicated in the meta-analyses by Chen et al. and Abargouei et al. [75,76]. A more beneficial effect of higher intakes of dietary protein may thus be evident if changes in fat mass are taken into consideration. Compliance with the intervention and study duration can also be expected to affect the observed efficacy. An overall effect of dietary protein is demonstrated in this meta-analysis, despite including 13 studies reporting poor compliance or not reporting compliance. Additionally, many of the studies evaluate good compliance based solely on self-reporting, which is known to be prone to give rise to bias due to over/underreporting [77]. Poor compliance may therefore be relevant in more of the studies, indicating that real effect size might be increased if these could be properly identified and excluded in the relevant subgroup analysis. In the primary meta-analysis, 19 of the studies show no significant difference in body weight change between the intervention and the control groups. Nevertheless, since the sensitivity analysis removing almost one third of the studies revealed comparable overall result, the potential biases of the studies is likely to have a limited effect the identified efficacy. In 11 of these studies, the mean duration of the intervention was 15 weeks, and the remaining eight studies were long-term interventions (≥24 weeks). This implies that a time factor may affect efficacy. The ability to detect an effect on body weight changes is reduced if the intervention is too short, whereas compliance may decrease over time in long intervention studies.

### 4.2. Personalized Dietary Approaches

Common to all the interventions in the studies included in this review is that, in order to increase dietary protein content, something else was decreased. In most cases, this was achieved through a relative decrease in carbohydrate. It is not possible to determine from these studies whether the effects seen can be attributed to an increased intake of protein or whether they are rather a result of a reduced intake of one of the other macronutrients. Especially, the reduction in carbohydrate intake may improve body weight management in individuals with compromised glucose control independently of the amount of protein consumed. Glucose response in the brain has been found to be diminished in individuals with obesity and poorly controlled type 2 diabetes mellitus compared to individuals of normal weight, which has been found to be related to low self-reported scores of satiety and fullness [78]. Additionally, in individuals of normal weight, an inverse association has been observed between postprandial insulin response and the subsequent ad libitum energy intake [20,79]. The effect on appetite has been suggested to be that the absorbed carbohydrate elevating the blood glucose needs to enter the brain (and possibly other tissues) before it can lead to satiety and reduce hunger. The findings suggest that postprandial glucostatic control may be important for control of appetite, and that reduced carbohydrate intake may have a beneficial effect on body weight management in individuals with compromised glucose control [17,80]. We do not know all details about the populations in the studies included in this meta-analysis, so the effect on body weight management may be masked by the fact that certain individuals perhaps responded strongly to the interventions while others did not. This is suggested by the associations between consumption of low glycemic load diets high in protein, fiber, and whole grains a with improved body weight management observed in individuals with prediabetes, but less so in individuals with normoglycemia (retrospective analyses by Ballesteros-Pomar et al. and Hjorth et al. [19,37,59]. The non-significant difference in responsiveness observed by Ballesteros-Pomar et al. is probably due to the lower number of individuals assessed (*n* = 36) as compared to the analyses by Hjorth et al. (*n* = 266) [37,59]. Thereby, HOMA-IR and fasting plasma glucose seem to be important biomarkers for successful dietary weight loss and weight loss maintenance. In individuals with compromised glucose control, body weight may be managed better if carbohydrate content of the diet is decreased, but we do not know whether carbohydrate should be replaced with protein or fat. This highlights the general limitation of such interventions, where substitutions are necessary to keep the energy content constant in order for the diets to be comparable [81]. Nevertheless, given that fat is less satiating than protein [5], we can still assume that substitution with protein instead of fat is more beneficial for body weight management. Findings show that the effect of increased dietary protein on body weight management is mediated by a lower energy density, suggesting that substitution with fat would be less beneficial. Furthermore, if only improvements in cardio-metabolic risk factors are taken into consideration, Hyde et al. recently showed that substituting carbohydrate with fat in isoenergetic diets benefits metabolic syndrome risk factors independent of whole-body or fat mass [82].

### 4.3. Validity of Results

The validity of our conclusions depends on the possible publication bias in this area. It has previously been shown that publication bias (the tendency to publish positive rather than negative findings) exists in the obesity treatment literature, and probably more so in observational studies than in randomized controlled trials [83,84]. However, we included only randomized controlled trials in the meta-analyses, and half of the studies identified did not show a significant difference in body weight change, so the effect identified from the primary meta-analysis is unlikely to be driven by publication bias. There was no indication of bias in the funnel plot, so the non-significant tendencies to asymmetry and small study effects identified by the Eggers and Begg tests, presumably caused by the similar SEM of most of the studies, can be assumed to be trustworthy [30]. Nevertheless, the results are limited by the fact that CI is imputed for most of the studies in the meta-analyses. However, it is important to note that this does not change the significance of the results for most of the studies. Furthermore, results are affected by the imputation in both directions, i.e., significant results are obtained despite non-significance being reported in the original paper and vice versa.

A general weakness of the studies is that the majority have a non-blinded design, as blinding is extremely difficult in studies applying full replacements in regular diets. If all foods are provided to the participants, at least a single-blinded design with blinding of the participants may be achievable, although this would be very time consuming and costly. Even so, such a study might not reflect a true effect of the diets in everyday life, as many of the foods will be pre-packed and perhaps prepared in advance to maintain the blinding. The non-blinded procedures in which the participants buy and cook the foods themselves, better represent the true effectiveness of the diets. However, compliance is known to increase when the foods are provided [34,48,56,64,66], which highlights a risk of bias in studies where substitutes are provided to the intervention group but not to the control group [33,68,70,71]. This creates a risk of a non-relevant control, since it is unknown whether the participants find substitutes that are relevant to compare with the items provided to the intervention group, and it may introduce difference in energy intake.

The effectiveness of increased dietary protein on body weight management must be interpreted in the light of other aspects. Further studies on the health benefits of different protein sources are needed before recommending increased intake of dietary protein. Protein sources should also be considered in terms of environmental footprint and socioeconomic availability. Potential differences in health benefits from different protein sources remain mainly unresolved, and little is known about the environmental footprint of increasing the production of different protein sources [85]. The price per gram protein varies considerably, dependent on the source, a factor that needs to be considered if recommendations are to be relevant for most of the population. Thereby, a holistic view is needed, balancing the potential metabolic benefits of increased dietary protein, the environmental impact of production, as well as affordability and accessibility of the diet.

## 5. Conclusions

The studies identified for this review suggest that diets rich in protein (ranging from 18–59 energy percentage (E%)) may have a beneficial effect on body weight management. However, there is insufficient evidence to determine the optimal level of protein based on this review. We did not find sufficient evidence to determine whether certain specific proteins have stronger effects than others. The effect sizes on group level may be limited, and the degree to which this approach is beneficial may be dependent on specific phenotypes, which indicates a need for more personalized approaches for prevention and treatment of overweight and obesity.

## Figures and Tables

**Table 1:** Strategy for the primary literature search conducted to investigate the evidence for the effect of dietary protein on body weight management.

	PubMed	Web of Science	Cochrane Library
Search (Body weight)	“dietary protein*” AND “body weight change*” OR “body weight management*” OR “body weight control*” OR “*weight loss*” OR “weight loss maintenance” OR “*weight regain*” NOT exercise* OR “physical activity*” OR *training* OR *pharma*	“dietary protein*” AND “body weight change*” OR “body weight management*” OR “body weight control*” OR “*weight loss*” OR “weight loss maintenance” OR “*weight regain*” NOT exercise* OR “physical activity*” OR *training* OR *pharma* AND human*	“dietary protein*” AND “body weight change*” OR “body weight management*” OR “body weight control*” OR “*weight loss*” OR “weight loss maintenance” OR “*weight regain*” NOT exercise* OR “physical activity*” OR *training* OR *pharma* AND human*
Filters	Humans; Clinical trial; Clinical study; Adult: 19–44 years; Middle Aged: 45–64 years	Article	Trials
Language restrictions	None	None	None
Search field	All fields	Topic	Title abstract keyword
Sort by	Most recent	Date (high to low)	Year first published—new to old

The systematic literature searches were conducted in PubMed, Web of Science, and Cochrane Library. Studies potentially eligible for inclusion and available as of 1 September 2021 were identified. *, Included to identify all derived word forms (e.g., dietary proteins); “x x”, Identifies only studies with the words combined (e.g. do not include studies only identified based on “dietary”, rather “dietary protein”.

**Table 2:** Key study features and participant characteristics of studies included in this systematic review.

Study Characteristic			Mean (Range)—Unless Indicated Otherwise
Protein vs. digestible CHO			
Number of studies			35
Number of participants			113 (12–773)
Male/Female			30 (0–168)/63 (0–300)
Age			46 (22–62) *
BMI			33.9 (29.9–45.6) *
Diet characteristics			
	Protein		
		HP	30 (20–59) E%
		SP	15 (2–20) E%
	Fat		
		HP	28 (5–30) E%
		SP	28 (3–30) E%
	CHO		
		HP	41 (25–48) E%
		SP	57 (50–95) E%
Number of studies evaluating effects on			
	Weight loss		18
	Weight loss maintenance		11
	Weight maintenance		6
**Protein vs. fiber**			
Number of studies			2
Number of participants			104 (83–125)
Male/Female			30 (0–59)/75 (66–83)
Age			41 (40–42) *
BMI			32.3 (31.5–34.0) *
Diet characteristics			See Supplementary Material Table S1
Number of studies evaluating effects on			
	Weight loss		0
	Weight loss maintenance		1
	Weight maintenance		1
**Protein vs. fat**			
Number of studies			2
Number of participants			75 (57–93)
Male/Female			13 (0–25)/63 (32–93)
Age			50 (mean only reported in one of the studies)
BMI			34.9 (34.0–35.7) *
Diet characteristics			
	Protein		
		HP	35 (30–40) E%
		HF	20 (20) E%
	Fat		
		HP	30 (30) E%
		HF	45 (40–50) E%
	CHO		
		HP	35 (30–40) E%
		HF	35 (30–40) E%
Number of studies evaluating effects on			
	Weight loss		1
	Weight loss maintenance		1
	Weight maintenance		0
**Protein supplementation vs. no supplementation (no placebo used)**			
Number of studies			2
Number of participants			134 (120–148)
Male/Female			NR
Age			44 (mean only reported in one of the studies)
BMI			29.5 (29.4–29.5) *
Diet characteristics			
	Amount of protein supplementation		39.1 (30.0–48.2) g/d
	Protein intake		
		I	18 (18) E%
		C	15 (15) E%
Number of studies evaluating effects on			
	Weight loss		0
	Weight loss maintenance		2
	Weight maintenance		0
**Different proteins—studies only included in separate meta-analysis**			
Number of studies			6
Number of participants			76 (18–131)
Male/Female			26 (0–68)/50 (15–95)
Age			45 (26–51) *
BMI			31. 4 (28.3–34.4) *
Diet characteristics			Number of studies
	Whey protein		3
	Lupin		1
	Animal vs. vegetable protein		1
	Specific peptide		1
Number of studies evaluating effects on			
	Weight loss		2
	Weight loss maintenance		2
	Weight maintenance		2

*, Range is based on means from the studies; BMI, body mass index; C, control group; CHO, carbohydrate, d, day; E%, energy percentage; HF, high fat (range: 40–50 E%); HP, high protein (range: 20–59 E%); I, intervention group; SP, standard protein (range: 2–20 E%).

## Supplementary Materials

The following are available online at https://www.mdpi.com/article/10.3390/nu13093193/s1, Table S1: Study features and participant characteristics of studies included in this systematic review; Table S2: Risk of bias assessment of studies included in in this systematic review; Figure S1: Funnel plot with one-time representation of all studies included in the primary meta-analysis; Figure S2: Meta-analysis of mean difference in body weight changes with 95% CI (kg) between exposures to various interventions with specific protein sources compared to matching controls.

## Abbreviations

BMI, body mass index; CI, confidence interval; E%, energy percentage; GI, gastrointestinal; MeSH, medical subject heading; HOMA-IR, homeostatic model assessment for insulin resistance; HP, high protein; ITT, intention-to-treat; PICOS, population, intervention, comparison, outcome and setting; PRISMA, preferred reporting items for systematic reviews and meta-analyses; SD, standard deviation; SEM, standard error of mean; SNP, single nucleotide polymorphism; TFAP2B, transcription factor AP-2 beta gene; SP, standard protein.
