# Supplementary material for: Are Dietary Proteins the Key to Successful Body Weight Management? A Systematic Review and Meta-Analysis of Studies Assessing Body Weight Outcomes after Interventions with Increased Dietary Protein
Source: Nutrients. 2021 Sep 14;13(9):3193. doi: 10.3390/nu13093193 (PMC8468854; doi:10.3390/nu13093193)
Supplement: Supplementary file 1 [file nutrients-13-03193-s001.zip › nutrients-1337225-supplementary.pdf]

Are dietary proteins the key to successful body weight management? A systematic review and meta-analyses of studies assessing body weight outcomes after interventions with increased dietary protein

Supplementary material

Results

Study characteristics

Table S1 Study features and participant characteristics of studies included in this systematic review

**Reference**<br><br>**Study name**<br>**(if stated)**	**Protein**<br>**Intervention type: amount of protein**	**Population**<br>**n (M/F),**<br>**Age (y),**<br>**BMI (kg/m^2)**	**Design**<br>**Type,**<br>**Length,**<br>**Compliance**	**Provision of foods**	**Potential commercial interest of funder**
**Protein *vs* digestible CHO**					
Abete *et al.* 2009[1]	Reduced total energy intake by 30%/d in relation to individual energy expenditure on the following diets:<br>HP: 30E% protein, 30E% fat, 40E% CHO *vs* SP: 17E%, 30E%, 53E%<br>(two different diets not relevant for this review were also included [n=16])	35 (35/0),<br>38±7,<br>31.8±3.0	NB P RCT,<br>8 wk,<br>Intake close to prescribed in both groups resulting in higher protein intake and lower CHO intake in HP group assessed by 3-d dietary records	Individual instructions from trained dieticians with weekly follow-up	No<br>(funded by the Government of Navarra)
Aldrich *et al.* 2011[2]	8 wk controlled food intake at app. 6800 kj/d + *ad libitum* diet for 12 wk on the following diets:<br>HP: 30E% protein, 30E% fat, 40E% CHO *vs* SP: 15E%, 30E%, 55E%<br>AND<br>HP with high whey protein: 30E% (15E% mixed protein/15E% whey protein, 30E%, 40E% *vs* SP	18 (3/15),<br>50±6,<br>30.3±1.6	NB P RCT,<br>20 wk,<br>Higher protein intake and lower CHO intake in HP groups compared to SP group assessed by daily questionnaires. Also, HP with high whey protein increased essential amino acid composition compared to HP group	First 8 wk: all foods were provided for all groups.<br>Last 12 wk: dietary instructions for all groups.<br>HP with high whey protein was additionally provided whey protein powder and pure protein bars	No<br>(funded by the National Centre for Research Resources, National Institutes of Health, US Department of Agriculture National Needs Graduate Fellowship Competitive, The National Institute of Food and agriculture, Next protein LLC)
Aller *et al.* 2014[3]<br><br>DIOGENES (same study as Larsen *et al.* 2010[4], but this paper reports 12-month results from two of the study centers)	8-wk LED resulting in ≥8% weight loss followed by 1 y weight loss maintenance on the following diets:<br>HP: 26E% protein, 28E% fat, 46E% CHO *vs* SP: 13E%, 28E%, 59E%<br>(a control group not relevant for this review was also included [n=52])	256 (103/153),<br>42±5,<br>29.9±4.1	NB P RCT,<br>1 y,<br>Intake close to prescribed in both groups resulting in higher protein intake and lower CHO intake in HP group assessed by nitrogen excretion in 24-h urine samples and 3-d dietary records	First 6 months of intervention: >80% of all relevant foods for each diet groups were provided through a lab-based shop at no cost.<br>Through whole intervention: Guidance by dieticians	No<br>(funded by the European Commission and food items provided free of charge. Funders were not involved in planning, execution or analyses of the study)

Are dietary proteins the key to successful body weight management? A systematic review and meta-analyses of studies assessing body weight outcomes after interventions with increased dietary protein

Supplementary material

**Reference**<br><br>**Study name**<br>**(if stated)**	**<u>Protein</u>**<br>**Intervention type: amount of protein**	**<u>Population</u>**<br>**n (M/F),**<br>**Age (y),**<br>**BMI (kg/m^2)**	**<u>Design</u>**<br>**Type,**<br>**Length,**<br>**Compliance**	**<u>Provision of foods</u>**	**<u>Potential commercial interest of funder</u>**
Baba<br>*et al.* 1999[5]	Hypoenergetic diets providing 80% of resting energy requirements on the following diets:<br>HP: 45E% protein, 30E% fat, 25E% CHO *vs* SP: 12E%, 30E%, 58E%	13 (13/0),<br>Adults,<br>>31.0	NB P RCT,<br>4 wk,<br>NA (enforced by weekly nutrition counselling)	7-d rotating standardized menus provided, and participants asked to consume the entire food basket	No<br>(funded by University Research Board, American University of Beirut and the Ousseimi Foundation, Geneva, Switzerland)
Baer<br>*et al.* 2011[6]	Inclusion of one of three supplements twice/d (providing a total of 1670 kJ/d) with no additional dietary guidance:<br>HP with whey protein: 59E% protein, 5E% fat, 36E% CHO *vs* SP: 2E%, 3E%, 95E%<br>AND<br>HP with soy protein: 57E%, 8E%, 35E% *vs* SP	73 (34/39),<br>51±9,<br>31.0±11.5	DB P RCT,<br>23 wk,<br>Compliance to two packets/d assessed by counting number of packets distributed and recounting those consumed	Supplements were provided	Maybe<br>(funded by the USDA and the US Whey Protein Research Consortium)
Ballesteros-Pomar<br>*et al.* 2009[7]<br><br>Phenotyping (not included in meta-analysis)	Total energy intake reduced by calculating the individually total energy requirement minus 4184 kJ/d on the following diets:<br>HP: 30E% protein, 30E% fat, 40E% CHO *vs* SP: 15E%, 30E%, 55E%	36 (12/24),<br>42±14,<br>32.6±2.3	NB P RCT,<br>16 wk,<br>75% of participants in HP group followed prescribed diet, 87% of participants in SP group followed prescribed diet assessed by daily food diaries	Dietary counselling and written material of diet	No<br>(no specific grant from any funding agency in the public, commercial or not-for-profit sectors)
Brinkworth<br>*et al.* 2004a[8]<br><br>(same study as Farnworth *et al.* 2003[9], but this paper reports results during weight loss maintenance)	12 wk energy restriction at ~6500 kJ/d, 4 wk energy balance, 1 y *ad libitum*, all periods with the following diets:<br>HP: 30E% protein, 30E% fat, 40E% CHO *vs* SP: 15E%, 30E%, 55E%	43 (12/31),<br>52±10,<br>34.1±3.9	NP P RCT,<br>1 y,<br>During the *ad libitum* weight loss maintenance phase, protein intake increased in SP group and decreased in HP group, resulting in no difference in protein or CHO between the groups assessed by urinary urea/creatinine ratio and food diaries	Dietary counselling	No<br>(funded by a National Health and Medical Research Grant and a Dairy Research and Development Corporation Grant)

Are dietary proteins the key to successful body weight management? A systematic review and meta-analyses of studies assessing body weight outcomes after interventions with increased dietary protein

Supplementary material

**Reference**<br><br>**Study name**<br>**(if stated)**	**Protein**<br>**Intervention type: amount of protein**	**Population**<br>**n (M/F),**<br>**Age (y),**<br>**BMI (kg/m^2)**	**Design**<br>**Type,**<br>**Length,**<br>**Compliance**	**Provision of foods**	**Potential commercial interest of funder**
Brinkworth<br>*et al.* 2004b[10]<br><br>(same study as Parker *et al.* 2002[11], but this paper reports results during weight loss maintenance)	12 wk energy restriction at ~6700 kJ/d, 4 wk energy balance, 1 y *ad libitum*, all periods with the following diets:<br>HP: 30E% protein, 30E% fat, 40E% CHO *vs* SP: 15E%, 30E%, 55E%	38 (15/23),<br>62±8,<br>33.5±5.5	NP P RCT,<br>1 y,<br>Increased protein intake in HP group and unchanged in SP group assessed by urinary urea/creatinine ratio indicating compliance with prescription in both groups	Dietary counselling	Maybe<br>(funded by Meadow Lea Foods, Mascot, NSW, Australia)
Campos-Nonato<br>*et al.* 2017[12]	Energy restriction with individually calculated deficit of ~-2000 kJ/d on the following diets:<br>HP: 20E% protein *vs* SP: 13E% protein<br>Fat and CHO: NR	118 (44/74),<br>44±11,<br>32.4±4.9	NB P RCT,<br>24 wk,<br>Questionnaire generating score of compliance on a 5-level Likert scale used, but results NR	Sample menus provided containing 15 different meal options (i.e. 5-d cycle of 3 meal options and 2 snacks/d).<br>HP group was additionally provided with replacements (shakes) and protein bars (as snack) made with soy protein.<br>SP group was additionally provided with conventional foods	Maybe<br>(NR)
Claessens<br>*et al.* 2009[13]	5-wk VLED followed by:<br>HP casein or HP whey: 25E% protein, 30E% fat, 45E% CHO *vs* SP: 15E%, 30E%, 55E%<br>AND<br>HP whey *vs* HP casein	48 (17/31),<br>45±9,<br>32.9±5.0	NB P RCR,<br>12 wk,<br>Higher protein intake and lower CHO intake in HP groups assessed by nitrogen excretion in 24-h urinary samples and 3-d dietary records	Dietary counselling.<br>HP casein and HP whey groups were additionally provided with intact casein or whey supplements to be consumed twice/d, respectively.<br>SP group were additionally provided with maltodextrin supplements to be consumed twice/d	No<br>(funded by Kerry Bio-Science, Almere)

Are dietary proteins the key to successful body weight management? A systematic review and meta-analyses of studies assessing body weight outcomes after interventions with increased dietary protein

Supplementary material

**Reference**<br><br>**Study name (if stated)**	**Protein**<br>**Intervention type: amount of protein**	**Population**<br>**n (M/F),**<br>**Age (y),**<br>**BMI (kg/m^2)**	**Design**<br>**Type,**<br>**Length,**<br>**Compliance**	**Provision of foods**	**Potential commercial interest of funder**
Clifton<br>*et al.* 2008[14]	12-wk weight loss followed by isoenergetic diets at 5600 kJ/d, both periods on the following diets:<br>HP: 34E% protein, 20E% fat, 46E% CHO *vs* SP: 17E%, 20E%, 64E%	79 (0/79),<br>50±10,<br>34.1±3.4	NP P RCT,<br>1 y,<br>Poor compliance resulting in only 3.6% higher protein intake in the HP group and no difference in CHO intake assessed by 3-d dietary records	Dietary counselling at individual dietician sessions	Maybe<br>(funded by a Medical Research grant from Meat and Livestock Australia)
Dalle Grave<br>*et al.* 2013[15]	Energy restriction at ~5000 kJ/d for women and ~6300 kJ/d for men on the following diets:<br>HP: 34E% protein, 20E% fat, 46E% CHO *vs* SP: 17E%, 20E%, 63E%	88 (37/51),<br>47±11,<br>45.6±6.7	NB P RCT,<br>1 y,<br>Adherence to dietary composition and energy intake evaluated to be ~3 on a maximum score of 4 (0=low compliance; 4=complete adherence) based on daily food diaries	Cognitive behavior therapy including guidelines for diet	Maybe<br>(NR)
Debridge<br>*et al.* 2009[16]	Energy restriction at ~2100-2300 kJ/d achieving weight loss of ≥10% followed by individually calculated energy requirements for weight loss maintenance on the following diets:<br>HP: 30E% protein, 30E% fat, 40E% CHO *vs* SP: 15E%, 30E%, 55E%	141 (70/71),<br>44±10,<br>39.0±6.7	NB P RCT,<br>1 y,<br>HP group consumed all macronutrients according to the recommended, whereas SP group consumed more protein and less CHO than recommended assessed by urea excretion in 24-h urine samples and 3-d dietary records	Monthly dietary counselling	Maybe<br>(NR)
Due<br>*et al.* 2004[17]	No energy restriction on the following diets:<br>HP: 25E% protein, 30E% fat, 45E% CHO *vs* SP: 12E%, 30E%, 58E%	50 (12/38),<br>40±11,<br>30.4±2.2	NB P RCT,<br>24 wk,<br>Higher protein intake and lower CHO intake in the HP group in accordance with prescription assessed by nitrogen excretion in 24-h urine samples and 7-d dietary records	All foods for each diet groups were provided through a lab-based shop at no cost	Maybe<br>(funded by the Danish Research and Development Programme for Food Technology, the Federation of Danish Pig Producers and Slaughterhouse, Danish Dairy Research Foundation, the Danish Livestock and Meat board. Foods provided for shop from various producers)

Are dietary proteins the key to successful body weight management? A systematic review and meta-analyses of studies assessing body weight outcomes after interventions with increased dietary protein

Supplementary material

**Reference**<br><br>**Study name (if stated)**	**Protein**<br>**Intervention type: amount of protein**	**Population**<br>**n (M/F),**<br>**Age (y),**<br>**BMI (kg/m^2)**	**Design**<br>**Type,**<br>**Length,**<br>**Compliance**	**Provision of foods**	**Potential commercial interest of funder**
Farnworth *et al.* 2003[9]<br><br>(same study as Brinkworth *et al.* 2004a[8], but this paper reports results during weight loss)	Energy restriction at ~6400 kJ/d + energy balance at ~8200 kJ/d, both periods on the following diets:<br>HP: 30E% protein, 30E% fat, 40E% CHO *vs* SP: 15E%, 30E%, 55E%	57 (14/43),<br>50±10,<br>34.1±3.7	NB P RCT,<br>16 wk,<br>Higher protein intake and lower CHO intake in HP group assessed by urinary urea/creatinine ratio and food diaries	Fixed menu plans with provision of key foods that made up 60% of energy intake	Maybe<br>(NR)
Flechtner-Mors *et al.* 2010[18]	Energy restriction with individually calculated deficit of ~-2000 kJ/d on the following diets:<br>HP: 30E% protein, 30E% fat, 40 E% CHO *vs* SP: 15E%, 30E%, 55E%	110 (22/88),<br>50±13,<br>36.3±4.7	NB P RCT,<br>1 y,<br>Higher protein intake and lower CHO intake in HP group assessed by 3-d dietary records	Dietary instructions according to allocations and provision of meal replacements and protein bars	No<br>(funded by the University of Ulm and Herbalife International Inc., who also provided meal replacements, snacks, and protein bars. The company had no role in data collection, analysis, or interpretation)
Griffin *et al.* 2013[19]	Energy restriction at ~5600 kJ/d on the following diets:<br>HP: 32E% protein, 25E% fat, 41E% CHO *vs* SP: 20E%, 21E%, 58E%	71 (0/71),<br>22±2,<br>34.0±4.2	SB P RCT,<br>1 y,<br>Intake close to prescription in both groups based on 3-d dietary records. However, tendency for a lower energy intake in the SP group compared to the HP group (difference: 1148 kJ)	NR	Maybe<br>(funded by Meat and Livestock Australia)
Hjorth *et al.* 2017[20]<br><br>DIOGENES – Phenotyping (not included in meta-analysis)	8-wk LED resulting in ≥8% weight loss followed by 1 y weight loss maintenance on the following diets:<br>HP: ~21E% protein, ~30E% fat, ~46E% CHO *vs* SP: ~17E%, ~30E%, ~51E%<br>(Recanalization of results from two of the diet groups included in DIOGENES)	266 (42/224),<br>42±7,<br>34.2±NR	NB P RCT,<br>1 y,<br>Intake close to prescribed in both groups resulting in higher protein intake and lower CHO intake in HP group assessed by nitrogen excretion in 24-h urine samples and 3-d dietary records	Dietary counselling with provision of recipes, cooking instructions and behavioral advice as well as provision of >80% of all relevant foods for each diet groups provided at two of eight study centers	Maybe<br>(funded by Gelesis Inc.)

Supplementary material

**Reference**<br><br>**Study name**<br>**(if stated)**	**Protein**<br>**Intervention type: amount of protein**	**Population**<br>**n (M/F),**<br>**Age (y),**<br>**BMI (kg/m^2)**	**Design**<br>**Type,**<br>**Length,**<br>**Compliance**	**Provision of foods**	**Potential commercial interest of funder**
Jesudason<br>*et al.* 2013[21]	Energy restriction at 6000 kJ/d (allowance of ≤7000 kJ/d for men) on the following diets:<br>HP: 30E% protein, 30E% fat, 40E% CHO *vs* SP: 20E%, 30E%, 50E%	45 (35/10),<br>61±2,<br>36.1	NB P RCT,<br>1 y,<br>HP was compliant to the prescription protein intake (90-120 g/d) whereas SP consumed more than the prescribed (55-70 g/d)	Diet-information booklets, food-selection guide, sample diet meal plan and a selection of diet-specific recipes provided	No<br>(funded by National Health and Medical Research Council Principal Research Fellowship)
Kasim-Karakas<br>*et al.* 2009[22]	Energy restricted by ~-3000 kJ/d on the following diets:<br>HP: 34E% protein, 26E% fat, 40E% CHO including supplementation with whey protein *vs* SP: 17E%, 26E%, 57E% including supplementation with glucose+maltodextrin	33 (0/33),<br>28±3,<br>36.3±1.4	DB P RCT,<br>8 wk,<br>NR	Uniform dietary guidelines and provision of powdered supplements providing 1000 kJ/d	Maybe<br>(funded by the California Dairy Foundation and the ALSAM foundation. Supplements provided by Glanbia Foods, Twin Falls, ID)
Krebs<br>*et al.* 2012[23]<br><br>The Diabetes Excess Weight Loss Trial (DEWL)	Energy restriction by -2000 kJ/d on the following diets:<br>HP: 30E% protein, 30E% fat, 40E% CHO *vs* SP: 15E%, 30E%, 55E%	419 (168/251),<br>58±10,<br>36.6±6.5	DB P RCT,<br>2 y,<br>None of the groups adhered to the prescribed for intake of protein or CHO	Group sessions with dietary counselling based on allocated diet as well as provision of portion charts, sample diet plans and recipes	No<br>(funded by the Health Research Council of New Zealand)
Labayen<br>*et al.* 2003[24]	Energy restriction with individually calculated deficit of ~-2100 kJ/d on the following diets:<br>HP: 30E% protein, 30E% fat, 40E% CHO *vs* SP: 15E%, 30E%, 55E%	11 (0/11),<br>42±6,<br>37.7±2.0	NB P RCT,<br>10 wk,<br>NR	Dietary counselling	Maybe<br>(NR)
Larsen<br>*et al.* 2010[4]<br><br>DIOGENES<br>(same study as Aller *et al.* 2014[3], but this study reports 26-wk follow-up data)	8-wk LED resulting in ≥8% weight loss followed by 2 y weight loss maintenance on the following diets:<br>HP: 26E% protein, 28E% fat, 46E% CHO *vs* SP: 13E%, 28E%, 59E%<br>(a control group not relevant for this review was also included [n=114])	773 (NR),<br>42±6,<br>NR	NB P RCT,<br>2 y,<br>Intake close to prescribed in both groups resulting in higher protein intake and lower CHO intake in HP group assessed by nitrogen excretion in 24-h urine samples and 3-d dietary records	Dietary instructions as well as provision of >80% of all relevant foods for each diet groups through a lab-based shop at no cost at two of eight study centers	No<br>(funded by the European Commission and food items provided free of charge. Funders were not involved in planning, execution or analyses of the study)

Supplementary material

**Reference**<br><br>**Study name**<br>**(if stated)**	**<u>Protein</u>**<br>**Intervention type: amount of protein**	**<u>Population</u>**<br>**n (M/F),**<br>**Age (y),**<br>**BMI (kg/m²)**	**<u>Design</u>**<br>**Type,**<br>**Length,**<br>**Compliance**	**<u>Provision of foods</u>**	**<u>Potential commercial interest of funder</u>**
Layman<br>*et al.* 2003[25]	No energy restriction on the following diets:<br>HP: 30E% protein, 30E% fat, 40E% CHO *vs* SP: 15E%, 30E%, 55E%	24 (0/24),<br>50±1,<br>30.3±1.0	NB P RCT,<br>10 wk,<br>Similar energy, fat and fiber intake in both groups and protein and CHO intake according to prescription assessed by 3-d dietary records	First 4 wk: all foods were provided in 2-wk diet rotation along with daily instructions by dietician<br>Remaining 6 wk: self-use of 2-wk diet rotations plan	Maybe<br>(funded by the Cattlemen's Beef Board, National Cattlemen's Beef Association, Kraft Foods, USDA/Hatch, and the Illinois Council on Food and Agriculture Research)
Layman<br>*et al.* 2009[26]	No energy restriction on the following diets:<br>HP: 30E% protein, 30E% fat, 40E% CHO *vs* SP: 15E%, 30E%, 55E%	130 (59/71),<br>45±14,<br>32.6±9.1	NB P RCT,<br>1 y,<br>Similar energy intake in both groups and protein and CHO intake according to prescription assessed by 24-h urinary urea, 3-d dietary records and triacylglycerol concentrations	Provision of menu plans according to allocation	Maybe<br>(funded by the National Cattlemen's Beef Association, Beef Checkoff and Kraft Foods)
Mahon<br>*et al.* 2007[27]	Energy restriction (app. 5000 kJ/d) of which app. 4000 kJ/d consisted of lacto-ovo vegetarian diet and app. 1000 kJ/d consisted of:<br>Beef (n=14) or chicken (n=15): 26E% protein, 26E% fat, 48E% CHO *vs* non-meat CHO/fat food items (n=14): 16E%, 26E%, 58E%<br>(a control group not relevant for this review was also included [n=11])	54 (0/54),<br>50-80,<br>25-34	NB P RCT,<br>9 wk,<br>All groups adhered to prescribed assessed by 24-h urinary urea/creatinine ratio and 3-d dietary records	Provision of dietary counselling, written instructions, menus and shopping lists as well as 1000 kJ/d of cooked beef, chicken or cookies according to allocation	Maybe<br>(funded by Cattlemen's Beef Board and the National Cattlemen's Beef Association, Agriculture Research Program & Lynn Fellowships at Purdue University, and National Institute of Health)
McAuley<br>*et al.* 2006[28]	16-wk weight loss followed by 36 wk weight loss maintenance on the following diets:<br>HP: 30E% protein, 30E% fat, 40E% CHO *vs* SP: 15E%, 30E%, 55E%<br>AND<br>HP *vs* HF: 20E%, 40E%, 40E%	93 (0/93),<br>30-70,<br>35.7±4.9	NB P RCT,<br>1 y,<br>Similar protein intake in HP and SP group and higher fat intake in HP group assessed by 3-d dietary records.<br>HF group consumed more CHO than prescribed	Dietary counselling	Maybe<br>(funded by the Health Council of New Zealand, a Bristol Myers Squibb Mead Johnson Unrestricted Research Grant)

**Reference**<br><br>**Study name**<br>**(if stated)**	**Protein**<br>**Intervention type: amount of protein**	**Population**<br>**n (M/F),**<br>**Age (y),**<br>**BMI (kg/m^2)**	**Design**<br>**Type,**<br>**Length,**<br>**Compliance**	**Provision of foods**	**Potential commercial interest of funder**
Noakes *et al.* 2005[29]	Energy restriction at 5600 kJ/d on the following diets:<br>HP: 34E% protein, 20E% fat, 46E% CHO *vs* SP: 17E%, 20E%, 63E%	100 (0/100),<br>50±10,<br>33.0±5.0	NB P RCT,<br>12 wk,<br>Similar energy intake in both groups as well as higher protein intake and lower CHO intake in HP group assessed by 24-h urinary urea/creatinine ratio and 3-d dietary records. However, fat intake was higher in the HP group	Dietary counselling as well as food-preparation sessions and provision of recipes	Maybe<br>(funded by a Medical research grant from Meat and Livestock Australia)
Parker *et al.* 2002[11]<br><br>(same study as Brinkworth *et al.* 2004b[10], but this paper reports results during weight loss)	12 wk energy restriction at ~6700 kJ/d, 4 wk energy balance, 1 y *ad libitum*, all periods on the following diets:<br>HP: 30E% protein, 30E% fat, 40E% CHO *vs* SP: 15E%, 30E%, 55E%	54 (19/35),<br>62±9,<br>34.1±5.2	NP P RCT,<br>16 wk,<br>Higher protein intake and lower CHO intake in HP group assessed by urinary urea/creatinine ratio and 3-d dietary records indicating compliance with prescription in both groups	Fixed menu plans with provision of key foods that made up 60% of energy intake	Maybe<br>(funded by Meadow Lea Foods)
Pedersen *et al.* 2014[30]	Energy restriction at 6000 kJ/d on the following diets:<br>HP: 30E% protein, 30E% fat, 40E% CHO *vs* SP: 20E%, 30E%, 50E%	45 (35/10),<br>61±9,<br>36.1±NR	NB P RCT,<br>1 y,<br>Higher protein in the HP group assessed by urea excretion in 24-h urine samples, daily food checklists and food frequency questionnaires	NR	Maybe<br>(NR)
Sargrad *et al.* 2005[31]	Energy restriction on the following diets:<br>HP: 27E% protein, 30E% fat, 43E% CHO *vs* SP: 19E%, 30E%, 51E%	12 (3/9),<br>48±9,<br>35.0±6.1	NB P RCT,<br>8 wk,<br>Similar energy intake in both groups as well as higher protein intake and lower CHO intake in HP group assessed by dietary recalls	Dietary counselling along with provision of printed material describing the diets according to allocation including exchange lists, serving sizes and sample menus	No<br>(funded by the National Institutes of Health grants and a Mentor-Based Training Grant from the American Diabetes Association)

**Reference**<br><br>**Study name (if stated)**	**Protein**<br>**Intervention type: amount of protein**	**Population**<br>**n (M/F),**<br>**Age (y),**<br>**BMI (kg/m^2)**	**Design**<br>**Type,**<br>**Length,**<br>**Compliance**	**Provision of foods**	**Potential commercial interest of funder**
Skov<br>*et al.* 1999[32]	No energy restriction on the following diets:<br>HP: 25E% protein, 30E% fat, 45E% CHO *vs* SP: 12E%, 30E%, 58E%	50 (12/38),<br>40±10,<br>30.5±2.0	NB P RCT,<br>24 wk,<br>Higher protein intake and lower CHO intake in HP group assessed by nitrogen excretion in 24-h urine samples and registrations of foods obtained from the shop plus registrations of additional foods consumed	All relevant foods for each diet groups were provided through a lab-based shop at no cost. Participants were asked to record potential additional foods consumed or left over from the shop food	Maybe<br>(funded by the Danish Research and Development Programme for Food Technology, the Federation of Danish Pig Producers and Slaughterhouses, Danish Dairy Research Foundation and the Danish Livestock and Meat Board)
Stocks<br>*et al.* 2013[33]<br><br>DIOGENES – Phenotyping (not included in meta-analysis)	8-wk LED resulting in ≥8% weight loss followed by 24 wk weight loss maintenance on the following diets:<br>HP: 26E% protein, 28E% fat, 46E% CHO *vs* SP: 13E%, 28E%, 59E%<br>(a control group not relevant for this review was also included [n=52])	468<br>(168/300),<br>42±6,<br>27-45	NB P RCT,<br>24 wk,<br>Intake close to prescribed in both groups resulting in higher protein intake and lower CHO intake in HP group assessed by nitrogen excretion in 24-h urine samples and 3-d dietary records	Dietary counselling with provision of recipes, cooking instructions and behavioral advice	No<br>(funded by the European Commission, the Nordea Foundation and the Danish strategic Research Council)
Sørensen<br>*et al.* 2012[34]	No energy restriction on the following diets:<br>HP: >40E% protein, 30E% fat, <30E% CHO *vs* SP: <15E%, 30E% fat, >55E% CHO	57 (0/57),<br>28±6,<br>30.6±8.2	NB P RCT,<br>24 wk,<br>Higher protein intake in HP group assessed by nitrogen excretion in 24-h urine samples and food diaries	Dietary counselling with dieticians and diet suggestions provided	Maybe<br>(funded by Foundation for Professional Development of Practices of Medical Specialists and the Danish Meat Association)
Tang<br>*et al.* 2013[35]	Energy restriction with individually calculated deficit of ~-3100 kJ/d on the following diets:<br>HP: 25E% protein, 25E% fat, 50E% CHO *vs* SP: 15E%, 25E%, 60E%	43 (43/0),<br>48±14,<br>31.7±3.3	NB P RCT,<br>12 wk,<br>Higher protein intake and lower CHO intake in HP group assessed by food check lists	Dietary counselling to follow 7-d menus according to allocation	Maybe<br>(funded by the National Pork Board, American Egg Board-Egg Nutrition Centre and Purdue Ingestive Behavior Research Centre)

Are dietary proteins the key to successful body weight management? A systematic review and meta-analyses of studies assessing body weight outcomes after interventions with increased dietary protein

Supplementary material

**Reference**<br><br>**Study name (if stated)**	**Protein**<br>**Intervention type: amount of protein**	**Population**<br>**n (M/F),**<br>**Age (y),**<br>**BMI (kg/m^2)**	**Design**<br>**Type,**<br>**Length,**<br>**Compliance**	**Provision of foods**	**Potential commercial interest of funder**
**Protein *vs* fiber**					
Reimer *et al.* 2017[36]	Two doses per day of isoenergetic (420 kJ) snack bars included as replacement for current snacks:<br>Protein (5 g whey protein) (n=29) *vs* fiber (6 g oligofructose + 2 g inulin) (n=29)<br>(a control group and a mixed group not relevant for this review were also included [n=67])	125 (59/66),<br>40±14,<br>31.5±5.5	DB P RCT,<br>12 wk,<br>NR	Bars were provided	Maybe<br>(funded by the General Mills Bell Institute of Nutrition)
Te Morenga *et al.* 2011[37]	Energy restricted by -2000-4000 kJ/d on the following diets:<br>HP: 30E% protein, 30E% fat, 40 E% CHO *vs* HFib: 20E%, 30E%, 50E% with emphasis on wholegrains and legumes	83 (0/83),<br>42±12,<br>34.0±4.9	SB P RCT,<br>8 wk,<br>Higher protein intake and lower CHO intake in HP group assessed by 3-d dietary records. However, fat intake was higher in HP group resulting in higher energy intake	Dietary counselling along with checklists, recipes and menu plans according to allocation. HP group was additionally provided with grocery vouchers with instructions to purchase lean protein foods. HFib group was additionally provided with six servings/d of key CHO food	No<br>(funded by Fonterra CO-operative Group Ltd, but they had no involvement in the design, interpretation or reporting of the data)
**Protein *vs* fat**					
McAuley *et al.* 2006[28]	Included under “Protein *vs* digestible CHO”				
Moran *et al.* 2005[38]	12 wk energy restriction at 6081 kJ/d followed by 4 wk energy balance at 7346 kJ/d, both periods on the following diets:<br>HP: 40E% protein, 30E% fat, 30E% CHO *vs* HF: 20E%, 50E%, 30E%	57 (25/32),<br>50±10,<br>34.0±3.5	NB P RCT,<br>16 wk,<br>All participants adhered to prescribed assessed by 24-h urinary urea/creatinine ratio and 3-d dietary records	Fortnightly meetings with dieticians for education on dietary regimen according to allocation	No<br>(funded by the National Health and Medical Research Council of Australia)
**Protein supplementation *vs* no supplementation (no placebo used)**					
Lejeune *et al.* 2005[39]	4 wk energy restriction followed by 16 wk weight loss maintenance on the following diets:<br>I: protein supplementation (30.0 g/d) *vs* C: no supplementation	120 (NR),<br>18-60,<br>29.4±2.6	NB P RCT,<br>20 wk,<br>Higher protein intake in the I group as assessed by nitrogen excretion in 24-h urine samples	Provision of additional protein for the I group.<br>Dietary counselling upon request for both groups	Maybe<br>(NR)

**Reference**<br><br>**Study name**<br>**(if stated)**	**<u>Protein</u>**<br>**Intervention type: amount of protein**	**<u>Population</u>**<br>**n (M/F),**<br>**Age (y),**<br>**BMI (kg/m^2)**	**<u>Design</u>**<br>**Type,**<br>**Length,**<br>**Compliance**	**<u>Provision of foods</u>**	**<u>Potential commercial interest of funder</u>**
Westerterp-Plantenga *et al.* 2004[40]	4 wk VLED followed by 12 wk weight loss maintenance on the following diets:<br>I: protein supplementation (48.2 g/d) *vs* C: no supplementation	148 (NR),<br>44±10,<br>29.5±2.5	NB P RCT,<br>16 wk,<br>Higher protein intake (18E% *vs* 15E%) in the I group as assessed by nitrogen excretion in 24-h urine samples	Provision of additional protein for the I group.<br>Dietary counselling for both groups	Maybe<br>(NR)
**Different proteins – studies only included in separate meta-analysis**					
Aldrich *et al.* 2011[2]	Included under "Protein *vs* digestible CHO"				
Baer *et al.* 2011[6]	Included under "Protein *vs* digestible CHO"				
Belski *et al.* 2011[41]	3 month energy restriction of ~-35%, 1 month stabilization and 8 month *ad libitum* diet. All periods with:<br>I: Foods enriched with lupin flour (incorporation rate of 25-40% by weight) *vs* C: Foods with wheat flour (Products were matched on energy, fat and sensory properties but differed in protein, CHO and fiber contents)	131 (68/63),<br>47±10,<br>31.4±2.8	DB P RCT,<br>1 y,<br>Higher protein and fiber intake in the lupin group compared to the control assessed by 3-d dietary records	Bread, biscuits and pasta provided based on flour according to allocation. Provided foods were consumed in place of other cereal-based products	No<br>(funded by the Western Australian Government, Department of Industry and Resources)
Claessens *et al.* 2009[13]	Included under "Protein *vs* digestible CHO"				

Are dietary proteins the key to successful body weight management? A systematic review and meta-analyses of studies assessing body weight outcomes after interventions with increased dietary protein

Supplementary material

**Reference**<br><br>**Study name (if stated)**	**<u>Protein</u>**<br>**Intervention type: amount of protein**	**<u>Population</u>**<br>**n (M/F),**<br>**Age (y),**<br>**BMI (kg/m^2)**	**<u>Design</u>**<br>**Type,**<br>**Length,**<br>**Compliance**	**<u>Provision of foods</u>**	**<u>Potential commercial interest of funder</u>**
Karamali *et al.* 2018[42]	Isoenergetic diets with 0.8 g protein/kg consisting of:<br>I: 35% animal proteins, 35% textured soy protein, 30% vegetable proteins *vs* C: 70% animal proteins, 30% vegetable proteins	60 (0/60),<br>26±6,<br>28.3±5.0	NB P RCT,<br>8 wk,<br>NR	Education regarding food preparations according to allocation and provision of textured soy protein in the I group	No<br>(funded by the Vice-chancellor for Research, IUMS and Iran)
Keogh *et al.* 2008[43]	Energy restriction including 2 sachets/d of:<br>I: glycomacropeptide-enriched whey protein isolate *vs* C: skim milk powder (Both products contained 15 g protein and 900 kJ/sachet)	127 (32/95),<br>50±12,<br>34.4±3.7	DB P RCT,<br>1 y,<br>NR (compliance was not different between groups, but data is not shown, and it is not reported whether they adhered to the prescribed)	Powder provided according to allocation	Maybe<br>(funded by Murray Goulburn Nutritionals, Victoria, Australia)

App., Approximately; BMI, Body mass index; C, Control group; CHO, Carbohydrate, d, Day; DP, Double-blinded; E%, Energy percentage; F, Female; h, Hour; HF, High fat; HFib, High fiber; HP, High protein; I, Intervention group; LED, Low energy diet; LF, Low fat; M, Male; NA, Not assessed, NB, non-blinded; NR, Not reported; P, Parallel, RCT, Randomized controlled trial; SB, Single-blinded; SP, Standard protein; VLED, Very low energy diet; wk; Week; y, Year

Are dietary proteins the key to successful body weight management? A systematic review and meta-analyses of studies assessing body weight outcomes after interventions with increased dietary protein

Supplementary material

Evaluation of risk of bias

Table S2 Risk of bias assessment of studies included in in this systematic review

**Reference**	**Random sequence generation (selection bias)**[1]	**Allocation concealment (selection bias)**[2]	**Blinding of participants and personnel (performance bias)**[3]	**Blinding of outcome assessment (detection bias)**[4]	**Incomplete outcome (body weight) data (attrition bias) (including if data from ITT analyses are available or not)**[5]	**Selective reporting (reporting bias) (reporting difference in body weight outcome between groups or not)**[6]	**Power calculation**[7]	**Drop out**[8]	**Compliance**[9]	**Explanations for high risk judgements**
**Protein *vs* digestible CHO**										
Abete *et al.* 2009[1]	Low	Unclear	High	Unclear	Low	Low	Low	Low	Low	- Non-blinded (not possible)
Aldrich *et al.* 2011[2]	Low	Unclear	High	Unclear	High	Low	Unclear	Low	Low	- Non-blinded (not possible)<br>- Excluding non-completers from analyses (however, only three participants dropped out, which was one from each of the three groups)
Aller *et al.* 2014[3]	Low	Low	High	Unclear	Low	Low	Low	Low	Low	- Non-blinded (not possible)
Baba *et al.* 1999[5]	Low	Unclear	High	Unclear	Low	Low	Unclear	Low	High	- Non-blinded (not possible)<br>- Compliance not assessed
Baer *et al.* 2011[6]	Low	Unclear	Low	Unclear	High	Low	Unclear	Low	Low	- Not analyzing data from all subjects entering the study
Ballesteros-Pomar *et al.* 2009[7]	Low	Low	High	Unclear	Not relevant	High	Not relevant	Low	High	- Non-blinded (not possible)<br>- P-value on difference in body weight outcome between groups not reported<br>- Difference in rates of compliance between the high protein group (75%) and the control group (87%)

Reference	Random sequence generation (selection bias)[1]	Allocation concealment (selection bias)[2]	Blinding of participants and personnel (performance bias)[3]	Blinding of outcome assessment (detection bias)[4]	Incomplete outcome (body weight) data (attrition bias) (including if data from ITT analyses are available or not)[5]	Selective reporting (reporting bias) (reporting difference in body weight outcome between groups or not)[6]	Power calculation[7]	Drop out[8]	Compliance[9]	Explanations for high risk judgements
Brinkworth *et al.* 2004a[8]	Low	Unclear	High	Unclear	High	Low	Unclear	High	High	- Non-blinded (not possible)<br>- Excluding non-completers from analyses<br>- 35% drop-out rate<br>- Poor compliance resulting in no difference in protein or CHO between the groups
Brinkworth *et al.* 2004b[10]	Low	Low	High	Unclear	High	Low	Unclear	High	Low	- Non-blinded (not possible)<br>- Excluding non-completers from analyses<br>- 42% drop-out rate
Campos-Nonato *et al.* 2017[12]	Low	Low	High	Unclear	High	Low	Low	Low	High	- Non-blinded (not possible)<br>- Excluding non-completers from analyses<br>- Compliance assessed but results not reported
Claessens *et al.* 2009[13]	Low	Unclear	High	Unclear	High	Low	Unclear	Low	Low	- Non-blinded (not possible)<br>- Excluding non-completers from analyses
Clifton *et al.* 2008[14]	Low	Unclear	High	Unclear	High	High	Unclear	High	High	- Non-blinded (not possible)<br>- Excluding non-completers from analyses<br>- P-value on difference in body weight outcome between groups not reported<br>- 34% drop-out rate<br>- Poor compliance resulting in only 3.6% difference in protein intake and no difference in CHO intake

Supplementary material

Reference	Random sequence generation (selection bias)[1]	Allocation concealment (selection bias)[2]	Blinding of participants and personnel (performance bias)[3]	Blinding of outcome assessment (detection bias)[4]	Incomplete outcome (body weight) data (attrition bias) (including if data from ITT analyses are available or not)[5]	Selective reporting (reporting bias) (reporting difference in body weight outcome between groups or not)[6]	Power calculation[7]	Drop out[8]	Compliance[9]	Explanations for high risk judgements
Dalle Grave *et al.* 2013[15]	Low	Low	High	Unclear	Low	Low	Low	Low	Low	- Non-blinded (not possible)
Debridge *et al.* 2009[16]	Low	Low	High	Unclear	Low	Low	Unclear	High	High	- Non-blinded (not possible)<br>- 42% drop-out rate<br>- Poor compliance in the control group resulting in lower difference in protein and CHO intake between the groups than prescribed
Due *et al.* 2004[17]	Low	Unclear	High	Unclear	High	Low	Unclear	Low	Low	- Non-blinded (not possible)<br>- Excluding non-completers from analyses
Farnworth *et al.* 2003[9]	High	Unclear	High	Unclear	High	High	Unclear	Low	Low	- Not described if group allocation was randomized<br>- Non-blinded (not possible)<br>- Excluding non-completers from analyses<br>- P-value on difference in body weight outcome between groups not reported
Flechtner-Mors *et al.* 2010[18]	Low	Unclear	High	Unclear	Low	Low	Unclear	High	Low	- Non-blinded (not possible)<br>- 27% drop-out rate
Griffen *et al.* 2013[19]	Low	Unclear	Low	Unclear	Low	Low	Low	High	Low	- 49% drop-out rate

Reference	Random sequence generation (selection bias)[1]	Allocation concealment (selection bias)[2]	Blinding of participants and personnel (performance bias)[3]	Blinding of outcome assessment (detection bias)[4]	Incomplete outcome (body weight) data (attrition bias) (including if data from ITT analyses are available or not)[5]	Selective reporting (reporting bias) (reporting difference in body weight outcome between groups or not)[6]	Power calculation[7]	Drop out[8]	Compliance[9]	Explanations for high risk judgements
Hjorth *et al.* 2017[20]	Low	Low	High	Unclear	Not relevant	Low	Not relevant	Low	Low	- Non-blinded (not possible)
Jesudason *et al.* 2013[21]	Low	Unclear	High	Unclear	High	High	Unclear	High	High	- Non-blinded (not possible)<br>- Excluding non-completers from analyses<br>- P-value on difference in body weight outcome between groups not reported<br>- 31% drop-out rate<br>- Poor compliance in the control group resulting in lower difference in protein intake between the groups than prescribed
Kasim-Karakas *et al.* 2009[22]	Low	Low	Low	Unclear	High	Low	Unclear	High	Unclear	- Excluding non-completers from analyses<br>- 27% drop-out rate
Krebs *et al.* 2012[23]	Low	Low	Low	Low	Low	Low	High	High	High	- 420 participants required to obtain 80% power assuming 12% drop-out, however, only 419 included and 30% dropped out<br>- 30% drop-out rate<br>- Poor compliance in both groups resulting in lower difference in protein and CHO intake between the groups than prescribed
Labayen *et al.* 2003[24]	Low	Unclear	High	Unclear	Unclear	Low	Unclear	Unclear	Unclear	- Non-blinded (not possible)

**Reference**	**Random sequence generation (selection bias)[1]**	**Allocation concealment (selection bias)[2]**	**Blinding of participants and personnel (performance bias)[3]**	**Blinding of outcome assessment (detection bias)[4]**	**Incomplete outcome (body weight) data (attrition bias) (including if data from ITT analyses are available or not)[5]**	**Selective reporting (reporting bias) (reporting difference in body weight outcome between groups or not)[6]**	**Power calculation[7]**	**Drop out[8]**	**Compliance[9]**	**Explanations for high risk judgements**
Larsen *et al.* 2010[4]	Low	Low	High	Unclear	Low	Low	Low	High	Low	- Non-blinded (not possible)<br>- 29% drop-out rate
Layman *et al.* 2003[25]	High	Unclear	High	Unclear	Unclear	Low	Unclear	Unclear	Low	- Not described if group allocation was randomized<br>- Non-blinded (not possible)
Layman *et al.* 2009[26]	Low	Unclear	High	Unclear	Low	Low	Low	Low*	Low	- Non-blinded (not possible)<br>*45% drop-out rate, but 50% was expected and thereby the desired 25 completers in each group was met resulting in 90% power
Mahon *et al.* 2007[27]	Low	Unclear	High	Unclear	High	Low	Low	Low	Low	- Non-blinded (not possible)<br>- Excluding non-completers from analyses
McAuley *et al.* 2006[28]	Low	Low	High	Unclear	High	Low	Low	Low	High	- Non-blinded (not possible)<br>- Excluding non-completers from analyses<br>- Poor compliance resulting in similar protein intake between groups and high fat intake in the high protein group than prescribed as well as higher CHO intake in the high fat group than prescribed
Noakes *et al.* 2005[29]	Low	Unclear	High	Unclear	High	Low	Unclear	Low	High	- Non-blinded (not possible)<br>- Excluding non-completers from analyses<br>- Higher fat intake than prescribed in the high protein group, however, this did not result in difference in energy intake

Reference	Random sequence generation (selection bias)[1]	Allocation concealment (selection bias)[2]	Blinding of participants and personnel (performance bias)[3]	Blinding of outcome assessment (detection bias)[4]	Incomplete outcome (body weight) data (attrition bias) (including if data from ITT analyses are available or not)[5]	Selective reporting (reporting bias) (reporting difference in body weight outcome between groups or not)[6]	Power calculation[7]	Drop out[8]	Compliance[9]	Explanations for high risk judgements
Parker *et al.* 2002[11]	Low	Unclear	High	Unclear	High	High	Unclear	Low	Low	- Non-blinded (not possible)<br>- Excluding non-completers from analyses<br>- P-value on difference in body weight outcome between groups not reported
Pedersen *et al.* 2014[30]	Low	Low	High	Low	High	Low	Unclear	High	Low	- Non-blinded (not possible)<br>- Excluding non-completers from analyses<br>- 41% drop-out rate
Sargrad *et al.* 2005[31]	Low	Unclear	High	Unclear	Unclear	Low	Unclear	Unclear	Low	- Non-blinded (not possible)
Skov *et al.* 1999[32]	Low	Unclear	High	Unclear	Unclear	Low	Unclear	Low	Low	- Non-blinded (not possible)
Stocks *et al.* 2013[33]	Low	Low	High	Unclear	Not relevant	Low	Not relevant	Low	Low	- Non-blinded (not possible)
Sørensen *et al.* 2012[34]	Low	Low	High	Unclear	Low	Low	Low	High	Low	- Non-blinded (not possible)<br>- 53% drop-out rate
Tang *et al.* 2013[35]	Low	Unclear	High	Unclear	High	Low	Unclear	Low	Low	- Non-blinded (not possible)<br>- Excluding non-completers from analyses
**Protein *vs* fiber**										

Reference	Random sequence generation (selection bias)[1]	Allocation concealment (selection bias)[2]	Blinding of participants and personnel (performance bias)[3]	Blinding of outcome assessment (detection bias)[4]	Incomplete outcome (body weight) data (attrition bias) (including if data from ITT analyses are available or not)[5]	Selective reporting (reporting bias) (reporting difference in body weight outcome between groups or not)[6]	Power calculation[7]	Drop out[8]	Compliance[9]	Explanations for high risk judgements
Reimer *et al.* 2017[36]	Low	Low	Low	Unclear	High	Low	Low	Low	Unclear	- Excluding non-completers from analyses
Te Morenga *et al.* 2011[37]	Low	Low	High	Unclear	High	Low	Low	Low	High	- Non-blinded (not possible)<br>- Excluding non-completers from analyses<br>- Higher fat intake in the high protein group resulted in higher energy intake
**Protein *vs* fat**										
McAuley *et al.* 2006[28]	Included under "Protein *vs* digestible CHO"									
Moran *et al.* 2005[38]	Low	Unclear	High	Unclear	High	Low	Unclear	High	Low	- Non-blinded (not possible)<br>- Excluding non-completers from analyses<br>- 28% drop-out rate

Are dietary proteins the key to successful body weight management? A systematic review and meta-analyses of studies assessing body weight outcomes after interventions with increased dietary protein

Supplementary material

Reference	Random sequence generation (selection bias)[1]	Allocation concealment (selection bias)[2]	Blinding of participants and personnel (performance bias)[3]	Blinding of outcome assessment (detection bias)[4]	Incomplete outcome (body weight) data (attrition bias) (including if data from ITT analyses are available or not)[5]	Selective reporting (reporting bias) (reporting difference in body weight outcome between groups or not)[6]	Power calculation[7]	Drop out[8]	Compliance[9]	Explanations for high risk judgements
**Protein supplementation *vs* no supplementation (no placebo used)**										
Lejeune *et al.* 2005[39]	Low	Unclear	High	Unclear	Unclear	Low	Unclear	Low	Low	- Non-blinded (not possible)
Westeterp-Plantenga *et al.* 2004[40]	Low	Unclear	High	Unclear	High	High	Unclear	Low	Low	- Non-blinded (not possible)<br>- Excluding non-completers from analyses<br>- P-value on difference in body weight outcome between groups not reported
**Different proteins – studies only included in separate meta-analysis**										
Aldrich *et al.* 2011[2]	Included under “Protein *vs* digestible CHO”									
Baer *et al.* 2011[6]	Included under “Protein *vs* digestible CHO”									
Belski *et al.* 2011[41]	Low	Low	Low	Unclear	Low	Low	Low	Low	Low	
Claessens *et al.* 2009[13]	Included under “Protein *vs* digestible CHO”									
Karamali *et al.* 2018[42]	Low	Low	High	Unclear	Low	Low	Low	Low	Unclear	- Non-blinded

Are dietary proteins the key to successful body weight management? A systematic review and meta-analyses of studies assessing body weight outcomes after interventions with increased dietary protein

Supplementary material

Reference	Random sequence generation (selection bias)[1]	Allocation concealment (selection bias)[2]	Blinding of participants and personnel (performance bias)[3]	Blinding of outcome assessment (detection bias)[4]	Incomplete outcome (body weight) data (attrition bias) (including if data from ITT analyses are available or not)[5]	Selective reporting (reporting bias) (reporting difference in body weight outcome between groups or not)[6]	Power calculation[7]	Drop out[8]	Compliance[9]	Explanations for high risk judgements
Keogh *et al.* 2008[43]	Low	Low	Low	Unclear	High	High	Unclear	High	Unclear	- Excluding non-completers from analyses<br>- P-value on difference in body weight outcome between groups not reported<br>- 43% drop-out rate

CHO, Carbohydrate; [1]Low risk = Randomized and allocation sufficiently described for replication; [2]Low risk = Allocation concealment described in detail; [3]Low risk = At least single-blinded (blinding of participants) during study performance; [4]Low risk = Data analyzed blinded on a first basis; [5]Low risk = Results reported from intention-to-treat analysis (100% of participants entering trial); [6]Low risk = Preferably mean (±standard deviation, standard error of mean or 95% confidence interval) on difference in body weight outcomes between groups are reported, but at least P-value on difference between groups and changes within each group allowing for calculation of mean difference between groups should be reported; [7]Low risk = Power calculation reported and sample size met according to predefined power calculation; [8]Low risk = ≤25% drop outs (up to 25% drop out is evaluated as expected as most of the studies include long-term interventions); [9]Low risk = Good compliance both in intervention and control group; High risk = Not fulfilling criteria for low risk; Unclear = No information reported; Not relevant = Not expected to introduce bias in results from this particular study if data are only used in secondary analyses assessing phenotyping

Effects of dietary proteins on body weight management

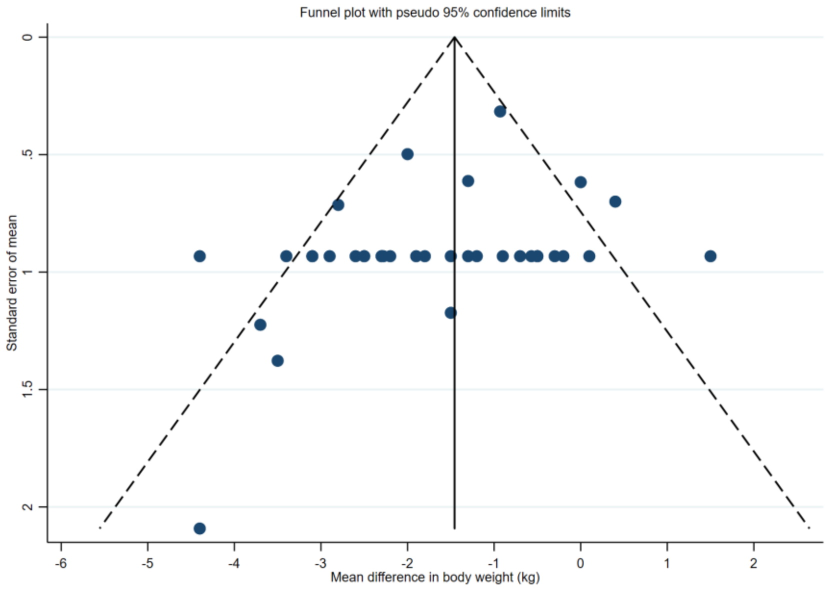

Figure S1 Funnel plot with one-time representation of all studies included in the primary meta-analysis (number of studies=37)

The funnel plot showed no sign of asymmetry. Slight tendencies for asymmetry (mean bias [95% CI]: -1.4 [-2.8; 0.1], P=0.06) as well as small-study effects (Kendall's tau: 1.8, P=0.07) for the studies included in the primary meta-analysis were indicated from the Eggers and Begg tests, respectively[44–46]

Are dietary proteins the key to successful body weight management? A systematic review and meta-analyses of studies assessing body weight outcomes after interventions with increased dietary protein

Supplementary material

Different effects from specific proteins

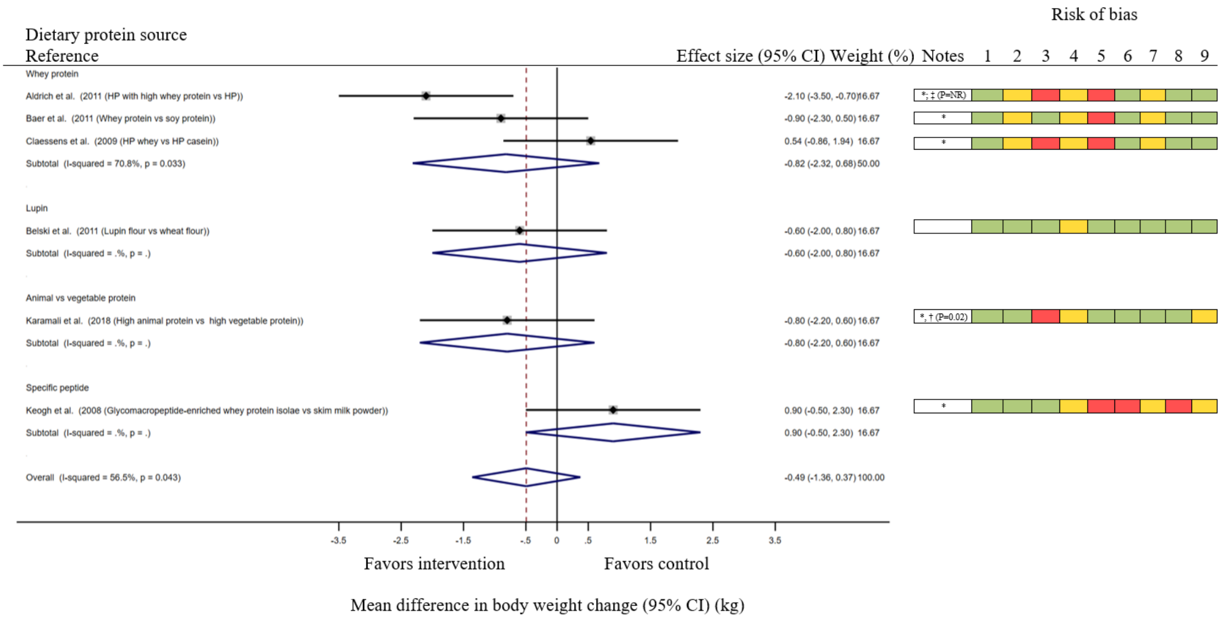

Figure S2 Meta-analysis of mean difference in body weight changes with 95% CI (kg) between exposures to various interventions with specific protein sources compared to matching controls
CI, Confidence interval; HP, High protein, NR, Not reported; SP, Standard protein; ∗, 95% CI imputed based on the average standard error of mean from the other studies[47]; †, Imputation of 95% CI causes non-significant difference despite significant difference being reported in the original paper; ‡, Imputation of 95% CI causes significant difference despite non-significant difference being reported in the original paper.
The meta-analysis is divided into different dietary protein sources. The grey marks around the mean from each study indicate the weight of the evidence from each study assessed in a random effects analysis. The blue diamonds summarize the total mean differences for each of the intervention categories, and finally for the overall result with width of the diamonds indicating the 95% CI.
The following criteria for risk of bias were assessed: 1. Random sequence generation (selection bias). 2. Allocation concealment (selection bias). 3. Blinding of participants and personnel (performance bias). 4. Blinding of outcome assessment (detection bias). 5. Incomplete outcome (body weight) data (attrition bias) (including if data from ITT analyses are available or not). 6. Selective reporting (reporting bias) (reporting differences in body weight outcome between groups or not). 7. Power calculation. 8. Drop out. 9. Compliance. Risk of bias was rated as “Low – indicated as green” or “High – indicated as red” according to predefined specifications (see Supplementary material Table 2 [legend]) or “Unclear – indicated as yellow” if no information on a potential bias was reported.

References

1. Abete, I.; Parra, D.; Martinez, J.A. Legume-, Fish-, or High-Protein-Based Hypocaloric Diets: Effects on Weight Loss and Mitochondrial Oxidation in Obese Men. *Journal of Medicinal Food* **2009**, *12*, 100–108, doi:10.1089/jmf.2007.0700.

2. Aldrich, N.D.; Reicks, M.M.; Sibley, S.D.; Redmon, J.B.; Thomas, W.; Raatz, S.K. Varying Protein Source and Quantity Do Not Significantly Improve Weight Loss, Fat Loss, or Satiety in Reduced Energy Diets among Midlife Adults. *Nutrition research* **2011**, *31*, 104-112, doi:10.1016/j.nutres.2011.01.004.

3. Aller, E.E.; Larsen, T.M.; Claus, H.; Lindroos, A.K.; Kafatos, A.; Pfeiffer, A.; Martinez, J.A.; Handjieva-Darlenska, T.; Kunesova, M.; Stender, S.; et al. Weight Loss Maintenance in Overweight Subjects on Ad Libitum Diets with High or Low Protein Content and Glycemic Index: The DIOGENES Trial 12-Month Results. *International journal of obesity* **2014**, *38*, 1511-1517, doi:10.1038/ijo.2014.52.

4. Larsen, T.M.; Dalskov, S.-M.; van Baak, M.; Jebb, S.A.; Papadaki, A.; Pfeiffer, A.F.H.; Martinez, J.A.; Handjieva-Darlenska, T.; Kunešová, M.; Pihlsgård, M. Diets with High or Low Protein Content and Glycemic Index for Weight-Loss Maintenance. *New England Journal of Medicine* **2010**, *363*, 2102–2113.

5. Baba, N.H.; Sawaya, S.; Torbay, N.; Habbal, Z.; Azar, S.; Hashim, S.A. High Protein vs High Carbohydrate Hypoenergetic Diet for the Treatment of Obese Hyperinsulinemic Subjects. *International journal of obesity and related metabolic disorders* **1999**, *23*, 1202-1206, doi:10.1038/sj.ijo.0801064.

6. Baer, D.J.; Stote, K.S.; Paul, D.R.; Harris, G.K.; Rumpler, W. V; Clevidence, B.A. Whey Protein but Not Soy Protein Supplementation Alters Body Weight and Composition in Free-Living Overweight and Obese Adults. *Journal of nutrition* **2011**, *141*, 1489-1494, doi:10.3945/jn.111.139840.

7. Ballesteros-Pomar, M.D.; Calleja-Fernández, A.R.; Vidal-Casariego, A.; Urioste-Fondo, A.M.; Cano-Rodríguez, I. Effectiveness of Energy-Restricted Diets with Different Protein: Carbohydrate Ratios: The Relationship to Insulin Sensitivity. *Public health nutrition* **2009**, *13*, 2119-2126, doi:10.1017/S1368980009991881.

8. Brinkworth, G.D.; Noakes, M.; Keogh, J.B.; Luscombe, N.D.; Wittert, G.A.; Clifton, P.M. (A) Long-Term Effects of a High-Protein, Low-Carbohydrate Diet on Weight Control and Cardiovascular Risk Markers in Obese Hyperinsulinemic Subjects. *International journal of obesity and related metabolic disorders* **2004**, *28*, 661-670, doi:10.1038/sj.ijo.0802617.

9. Farnsworth, E.; Luscombe, N.D.; Noakes, M.; Wittert, G.; Argyiou, E.; Clifton, P.M. Effect of a High-Protein, Energy-Restricted Diet on Body Composition, Glycemic Control, and Lipid Concentrations in Overweight and Obese Hyperinsulinemic Men and Women. *American journal of clinical nutrition* **2003**, *78*, 31-39, doi:10.1093/ajcn/78.1.31.

10. Brinkworth, G.D.; Noakes, M.; Parker, B.; Foster, P.; Clifton, P.M. (B) Long-Term Effects of Advice to Consume a High-Protein, Low-Fat Diet, Rather than a Conventional Weight-Loss Diet, in Obese Adults with Type 2 Diabetes: One-Year Follow-up of a Randomised Trial. *Diabetologia* **2004**, *47*, 1677-1686, doi:10.1007/s00125-004-1511-7.

11. Parker, B.; Noakes, M.; Luscombe, N.; Clifton, P. Effect of a High-Protein, High-Monounsaturated Fat Weight Loss Diet on Glycemic Control and Lipid Levels in Type 2 Diabetes. *Diabetes Care* **2002**, *25*, 425–430, doi:10.2337/diacare.25.3.425.

12. Campos-Nonato, I.; Hernandez, L.; Barquera, S. Effect of a High-Protein Diet versus Standard-Protein Diet on Weight Loss and Biomarkers of Metabolic Syndrome: A Randomized Clinical Trial. *Obesity facts* **2017**, *10*, 238-251, doi:10.1159/000471485.

13. Claessens, M.; van Baak, M.A.; Monsheimer, S.; Saris, W.H. The Effect of a Low-Fat, High-Protein or High-Carbohydrate Ad Libitum Diet on Weight Loss Maintenance and Metabolic Risk Factors. *International journal of obesity* **2009**, *33*, 296-304, doi:10.1038/ijo.2008.278.

14. Clifton, P.M.; Keogh, J.B.; Noakes, M. Long-Term Effects of a High-Protein Weight-Loss Diet. *American Journal of Clinical Nutrition* **2008**, *87*, 23–29, doi:10.1093/ajcn/87.1.23.

15. Dalle Grave, R.; Calugi, S.; Gavasso, I.; El Ghoch, M.; Marchesini, G. A Randomized Trial of Energy-Restricted High-Protein versus High-Carbohydrate, Low-Fat Diet in Morbid Obesity. *Obesity* **2013**, *21*, 1774-1781, doi:10.1002/oby.20320.

16. Delbridge, E.A.; Prendergast, L.A.; Pritchard, J.E.; Proietto, J. One-Year Weight Maintenance after Significant Weight Loss in Healthy Overweight and Obese Subjects: Does Diet Composition Matter? *American journal of clinical nutrition* **2009**, *90*, 1203-1214, doi:10.3945/ajcn.2008.27209.

17. Due, A.; Toubro, S.; Skov, A.R.; Astrup, A. Effect of Normal-Fat Diets, Either Medium or High in Protein, on Body Weight in Overweight Subjects: A Randomised 1-Year Trial. *International journal of obesity and related metabolic disorders* **2004**, *28*, 1283-1290, doi:10.1038/sj.ijo.0802767.

18. Flechtner-Mors, M.; Boehm, B.O.; Wittmann, R.; Thoma, U.; Ditschuneit, H.H. Enhanced Weight Loss with Protein-Enriched Meal Replacements in Subjects with the Metabolic Syndrome. *Diabetes/metabolism research and reviews* **2010**, *26*, 393-405, doi:10.1002/dmrr.1097.

19. Griffin, H.J.; Cheng, H.L.; O'Connor, H.T.; Rooney, K.B.; Petocz, P.; Steinbeck, K.S. Higher Protein Diet for Weight Management in Young Overweight Women: A 12-Month Randomized Controlled Trial. *Diabetes, obesity & metabolism* **2013**, *15*, 572-575, doi:10.1111/dom.12056.

20. Hjorth, M.F.; Ritz, C.; Blaak, E.E.; Saris, W.H.; Langin, D.; Poulsen, S.K.; Larsen, T.M.; Sørensen, T.I.; Zohar, Y.; Astrup, A. Pretreatment Fasting Plasma Glucose and Insulin Modify Dietary Weight Loss Success: Results from 3 Randomized Clinical Trials. *The American Journal of Clinical Nutrition* **2017**, *106*, 499–505, doi:10.3945/ajcn.117.155200.

21. Jesudason, D.R.; Pedersen, E.; Clifton, P.M. Weight-Loss Diets in People with Type 2 Diabetes and Renal Disease: A Randomized Controlled Trial of the Effect of Different Dietary Protein Amounts. *American journal of clinical nutrition* **2013**, *98*, 494-501, doi:10.3945/ajcn.113.060889.

22. Kasim-Karakas, S.E.; Almario, R.U.; Cunningham, W. Effects of Protein versus Simple Sugar Intake on Weight Loss in Polycystic Ovary Syndrome (According to the National Institutes of Health Criteria). *Fertility and sterility* **2009**, *92*, 262-270, doi:10.1016/j.fertnstert.2008.05.065.

23. Krebs, J.D.; Elley, C.R.; Parry-Strong, A.; Lunt, H.; Drury, P.L.; Bell, D.A.; Robinson, E.; Moyes, S.A.; Mann, J.I. The Diabetes Excess Weight Loss (DEWL) Trial: A Randomised Controlled Trial of High-Protein versus High-Carbohydrate Diets over 2 Years in Type 2 Diabetes. *Diabetologia* **2012**, *55*, 905-914, doi:10.1007/s00125-012-2461-0.

24. Labayen, I.; Díez, N.; González, A.; Parra, D.; Martínez, J.A. Effects of Protein vs. Carbohydrate-Rich Diets on Fuel Utilisation in Obese Women during Weight Loss. *Forum of nutrition* **2003**, *56*, 168-170.

25. Layman, D.K.; Boileau, R.A.; Erickson, D.J.; Painter, J.E.; Shiue, H.; Sather, C.; Christou, D.D. Human Nutrition and Metabolism A Reduced Ratio of Dietary Carbohydrate to Protein Improves Body Composition and Blood Lipid Profiles during Weight Loss in Adult Women 1,2. *American Society for Nutritional Sciences* **2003**, *133*, 411–417.

26. Layman, D.K.; Evans, E.M.; Erickson, D.; Seyler, J.; Weber, J.; Bagshaw, D.; Griel, A.; Psota, T.; Kris-Etherton, P. A Moderate-Protein Diet Produces Sustained Weight Loss and Long-Term Changes in Body Composition and Blood Lipids in Obese Adults. *Journal of nutrition* **2009**, *139*, 514-521, doi:10.3945/jn.108.099440.

27. Mahon, A.K.; Flynn, M.G.; Stewart, L.K.; McFarlin, B.K.; Iglay, H.B.; Mattes, R.D.; Lyle, R.M.; Considine, R. V; Campbell, W.W. Protein Intake during Energy Restriction: Effects on Body Composition and Markers of Metabolic and Cardiovascular Health in Postmenopausal Women. *Journal of the American College of Nutrition* **2007**, *26*, 182-189, doi:10.1080/07315724.2007.10719600.

28. McAuley, K.A.; Smith, K.J.; Taylor, R.W.; McLay, R.T.; Williams, S.M.; Mann, J.I. Long-Term Effects of Popular Dietary Approaches on Weight Loss and Features of Insulin Resistance. *International journal of obesity* **2006**, *30*, 342-349, doi:10.1038/sj.ijo.0803075.

29. Noakes, M.; Keogh, J.B.; Foster, P.R.; Clifton, P.M. Effect of an Energy-Restricted, High-Protein, Low-Fat Diet Relative to a Conventional High-Carbohydrate, Low-Fat Diet on Weight Loss, Body Composition, Nutritional Status, and Markers of Cardiovascular Health in Obese Women. *American journal of clinical nutrition* **2005**, *81*, 1298-1306, doi:10.1093/ajcn/81.6.1298.

30. Pedersen, E.; Jesudason, D.R.; Clifton, P.M. High Protein Weight Loss Diets in Obese Subjects with Type 2 Diabetes Mellitus. *Nutrition, metabolism, and cardiovascular diseases* **2014**, *24*, 554-562, doi:10.1016/j.numecd.2013.11.003.

31. Sargrad, K.R.; Homko, C.; Mozzoli, M.; Boden, G. Effect of High Protein vs High Carbohydrate Intake on Insulin Sensitivity, Body Weight, Hemoglobin A1c, and Blood Pressure in Patients with Type 2 Diabetes Mellitus. *Journal of the american dietetic association* **2005**, *105*, 573-580, doi:10.1016/j.jada.2005.01.009.

32. Skov, A.R.; Toubro, S.; Rønn, B.; Holm, L.; Astrup, A. Randomized Trial on Protein vs Carbohydrate in Ad Libitum Fat Reduced Diet for the Treatment of Obesity. *International journal of obesity and related metabolic disorders* **1999**, *23*, 528-536, doi:10.1038/sj.ijo.0800867.

33. Stocks, T.; Ängquist, L.; Hager, J.; Charon, C.; Holst, C.; Martinez, J.A.; Saris, W.H.; Astrup, A.; Sørensen, T.I.; Larsen, L.H. TFAP2B -Dietary Protein and Glycemic Index Interactions and Weight Maintenance after Weight Loss in the DiOGenes Trial. *Human heredity* **2013**, *75*, 213-219, doi:10.1159/000353591.

34. Sorensen, L.B.; Soe, M.; Halkier, K.H.; Stigsby, B.; Astrup, A. Effects of Increased Dietary Protein-to-Carbohydrate Ratios in Women with Polycystic Ovary Syndrome. *The American journal of clinical nutrition* **2012**, *95*, 39–48, doi:10.3945/ajcn.111.020693.

35. Tang, M.; Armstrong, C.L.; Leidy, H.J.; Campbell, W.W. Normal vs. High-Protein Weight Loss Diets in Men: Effects on Body Composition and Indices of Metabolic Syndrome. *Obesity* **2013**, *21*, 204-210, doi:10.1002/oby.20078.

36. Reimer, R.A.; Willis, H.J.; Tunnicliffe, J.M.; Park, H.; Madsen, K.L.; Soto-Vaca, A. Inulin-Type Fructans and Whey Protein Both Modulate Appetite but Only Fructans Alter Gut Microbiota in Adults with Overweight/Obesity: A Randomized Controlled Trial. *Molecular nutrition & food research* **2017**, *61*, 1–12, doi:10.1002/mnfr.201700484.

37. Te Morenga, L.A.; Levers, M.T.; Williams, S.M.; Brown, R.C.; Mann, J. Comparison of High Protein and High Fiber Weight-Loss Diets in Women with Risk Factors for the Metabolic Syndrome: A Randomized Trial. *Nutrition journal* **2011**, *10*, 1–9, doi:10.1186/1475-2891-10-40.

38. Moran, L.J.; Luscombe-Marsh, N.D.; Noakes, M.; Wittert, G.A.; Keogh, J.B.; Clifton, P.M. The Satiating Effect of Dietary Protein Is Unrelated to Postprandial Ghrelin Secretion. *Journal of clinical endocrinology and metabolism* **2005**, *90*, 5205-5211, doi:10.1210/jc.2005-0701.

39. Lejeune, M.P.; Kovacs, E.M.; Westerterp-Plantenga, M.S. Additional Protein Intake Limits Weight Regain after Weight Loss in Humans. *British journal of nutrition* **2005**, *93*, 281-289, doi:10.1079/bjn20041305.

40. Westerterp-Plantenga, M.S.; Lejeune, M.P.G.M.; Nijs, I.; Van Ooijen, M.; Kovacs, E.M.R. High Protein Intake Sustains Weight Maintenance after Body Weight Loss in Humans. *International Journal of Obesity* **2004**, *28*, 57–64, doi:10.1038/sj.ijo.0802461.

41. Belski, R.; Mori, T.A.; Puddey, I.B.; Sipsas, S.; Woodman, R.J.; Ackland, T.R.; Beilin, L.J.; Dove, E.R.; Carlyon, N.B.; Jayaseena, V.; et al. Effects of Lupin-Enriched Foods on Body Composition and Cardiovascular Disease Risk Factors: A 12-Month Randomized Controlled Weight Loss Trial. *International journal of obesity* **2011**, *35*, 810-819, doi:10.1038/ijo.2010.213.

42. Karamali, M.; Kashanian, M.; Alaeinasab, S.; Asemi, Z. The Effect of Dietary Soy Intake on Weight Loss, Glycaemic Control, Lipid Profiles and Biomarkers of Inflammation and Oxidative Stress in Women with Polycystic Ovary Syndrome: A Randomised Clinical Trial. *Journal of human nutrition and dietetics* **2018**, *31*, 533-543, doi:10.1111/jhn.12545.

43. Keogh, J.B.; Clifton, P. The Effect of Meal Replacements High in Glycomacropeptide on Weight Loss and Markers of Cardiovascular Disease Risk. *American journal of clinical nutrition* **2008**, *87*, 1602-1605, doi:10.1093/ajcn/87.6.1602.

44. Begg, C.B.; Mazumdar, M. Operating Characteristics of a Rank Correlation Test for Publication Bias. *Biometrics* **1994**, *50*, 1088–1101, doi:10.2307/2533446.

45. Egger, M.; Smith, G.D.; Schneider, M.; Minder, C. Bias in Meta-Analysis Detected by a Simple , Graphical Test Measures of Funnel Plot Asymmetry. *Bmj* **1997**, *315*, 629–634, doi:10.1136/bmj.315.7109.629.

46. Sterne, J.A.C.; Sutton, A.J.; Ioannidis, J.P.A.; Terrin, N.; Jones, D.R.; Lau, J.; Carpenter, J.; Rücker, G.; Harbord, R.M.; Schmid, C.H.; et al. Recommendations for Examining and Interpreting Funnel Plot Asymmetry in Meta-Analyses of Randomised Controlled Trials. *Bmj* **2011**, *343*, 1–8, doi:10.1136/bmj.d4002.

47. Deeks JJ, Higgins JPT, A.D. (editors). Chapter 10: Analysing Data and Undertaking Meta-Analyses. In: Higgins JPT, Thomas J, Chandler J, Cumpston M, Li T, Page MJ, Welch VA (Editors). Cochrane Handbook for Systematic Reviews of Interventions Version 6.0 (Updated July 2019). Available online: https://training.cochrane.org/handbook/current/chapter-10 (accessed on 3 March 2020).
